# Single-nucleus sequencing reveals enriched expression of genetic risk factors in extratelencephalic neurons sensitive to degeneration in ALS

**DOI:** 10.1038/s43587-024-00640-0

**Published:** 2024-06-21

**Authors:** Francesco Limone, Daniel A. Mordes, Alexander Couto, Brian J. Joseph, Jana M. Mitchell, Martine Therrien, Sulagna Dia Ghosh, Daniel Meyer, Yingying Zhang, Melissa Goldman, Laura Bortolin, Inma Cobos, Beth Stevens, Steven A. McCarroll, Irena Kadiu, Aaron Burberry, Olli Pietiläinen, Kevin Eggan

**Affiliations:** 1 Department of Stem Cell and Regenerative Biology, Harvard University, Cambridge, MA USA; 2 Stanley Center for Psychiatric Research, Broad Institute of MIT and Harvard, Cambridge, MA USA; 3 Neuroscience Institute, NYU Grossman School of Medicine, New York, NY USA; 4 Department of Pathology, Massachusetts General Hospital, Boston, MA USA; 5 FM Kirby Neurobiology Center, Boston Children’s Hospital, Boston, MA USA; 6 Department of Genetics, Harvard Medical School, Boston, MA USA; 7 Howard Hughes Medical Institute, Boston, MA USA; 8 Neuroinflammation Focus Area, UCB Pharma, Braine-l’Alleud, Belgium; 9 Department of Pathology, School of Medicine, Case Western Reserve University, Cleveland, OH USA; 10 Neuroscience Center, Helsinki Institute of Life Science, University of Helsinki, Helsinki, Finland

**Keywords:** Neuroimmunology, Amyotrophic lateral sclerosis, Oligodendrocyte, Motor cortex, Ageing

## Abstract

Amyotrophic lateral sclerosis (ALS) is a neurodegenerative disorder characterized by a progressive loss of motor function linked to degenerating extratelencephalic neurons/Betz cells (ETNs). The reasons why these neurons are selectively affected remain unclear. Here, to understand the unique molecular properties that may sensitize ETNs to ALS, we performed RNA sequencing of 79,169 single nuclei from cortices of patients and controls. In both patients and unaffected individuals, we found significantly higher expression of ALS risk genes in *THY1*^+^ ETNs, regardless of diagnosis. In patients, this was accompanied by the induction of genes involved in protein homeostasis and stress responses that were significantly induced in a wide collection of ETNs. Examination of oligodendroglial and microglial nuclei revealed patient-specific downregulation of myelinating genes in oligodendrocytes and upregulation of an endolysosomal reactive state in microglia. Our findings suggest that selective vulnerability of extratelencephalic neurons is partly connected to their intrinsic molecular properties sensitizing them to genetics and mechanisms of degeneration.

## Abstract (web summary)

Using single-nucleus RNA sequencing data from patients with sporadic amyotrophic lateral sclerosis (ALS) cortices, the authors find that higher expression of ALS risk genes is accompanied by upregulation of stress responses in groups of extratelencephalic neurons. Analyses of glial nuclei revealed a downregulation of myelination genes in oligodendrocytes and upregulation of reactive state genes in microglia.

## Main

Amyotrophic lateral sclerosis (ALS) is a neuromuscular disease with survival limited to 2–5 years from onset, the most common motor neuron disease in aging and the neurodegenerative disease with one of the earliest onsets, in the mid-to-late 50s^1^. Although specific genetic causes have been identified, most cases are sporadic (~90%), have no family history and unknown etiology^2^, thus rendering modeling of the disease difficult^3^. Variants in genes associated with ALS can contribute to a related disorder, frontotemporal dementia (FTD), leading to the view of ALS and FTD as clinical manifestations of shared molecular causes. Bulk RNA sequencing of ALS postmortem brains have identified differences^4^ and similarities between sporadic and familial^5^ cases and highlighted shared profiles^6–8^. While they provided valuable insights, these studies had limited resolution on the cell types altered by ALS.

The most striking feature in ALS–FTD are protein aggregates of TAR DNA/RNA-binding protein 43 (TDP-43) in over 95% of ALS cases and ~50% of FTD cases, mostly in neurons^9^, providing one shared mechanism. It is still unknown how familial mutations and sporadic onset might converge on the formation of these aggregates and how it specifically affects classes of extratelencephalic corticospinal motor neurons (CSMNs), that is, Betz^10^ and von Economo cells^11^. Moreover, strong evidence demonstrated that cells other than neurons are key mediators of disease progression and it remains unclear how these might contribute to the disease^7,12,13^.

Methods to study heterogeneity at a single-cell level have rapidly advanced and their application to human postmortem brain tissue is beginning to emerge. In this Article, we applied single-nucleus RNA sequencing (snRNAseq) and in vitro human induced pluripotent stem cell modeling to investigate changes in cortical cell types in sporadic ALS (sALS). Our profiling identified the intrinsically higher expression of ALS–FTD risk factors in a specific class of extratelencephalic excitatory neurons. In patients with ALS, these neurons and other subclasses of ETNs selectively express higher levels of genes connected to unfolded protein responses and RNA metabolism. We found that excitatory neuronal vulnerability is accompanied by a decrease in myelination-related transcripts in oligodendroglial cells and an upregulation of a reactive, proinflammatory state in microglia. We provide a preliminary, insightful view of disruptions triggered in human motor cortices in ALS and implicate aging-associated mechanisms that link altered proteostasis and inflammation to specific cell types in ALS.

## Results

### snRNAseq profiling of ALS cortex

**Fig. 1:**
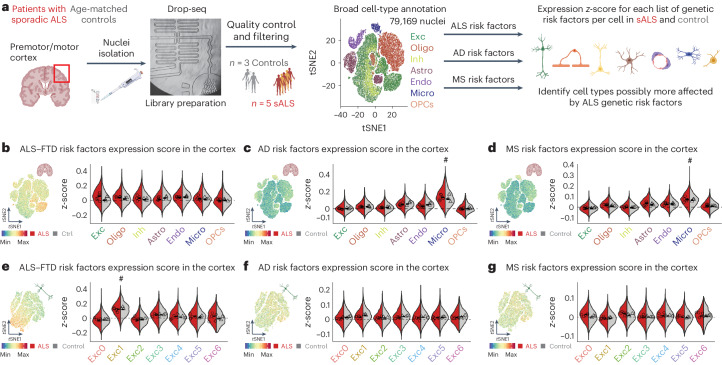
Cellular susceptibility to ALS–FTD in the human postmortem cortex. **a**, Diagram of the workflow for isolation of nuclei from cortices of patients with ALS and age-matched controls followed by snRNAseq and assessment of expression of gene modules associated with neurodegenerative diseases. Exc, excitatory neurons; Inh, inhibitory neurons; Oligo, oligodendrocytes; OPCs, oligodendrocyte progenitor cells; Micro, microglia; Astro, astrocytes; Endo, endothelial cells. **b**–**d**, *t*SNE projections and violin plots of *z*-scores for expression of genes associated with ALS–FTD (**b**), AD (**c**) and MS (**d**) in the different cell types identified (the bars denote median for each side of the violin and the symbols indicate the average score per individual). **e**–**g**, *t*SNE projections and violin plots of *z*-scores for expression of genes associated with the ALS–FTD (**e**), AD (**f**) and MS (**g**) in the different subtypes of excitatory neurons (the bars denote median for each side of the violin and the symbols indicate the average score per individual) (in **a**–**g**, *n* = 3 control individuals and *n* = 5 patients with sALS).

**Extended Data Fig. 1:**
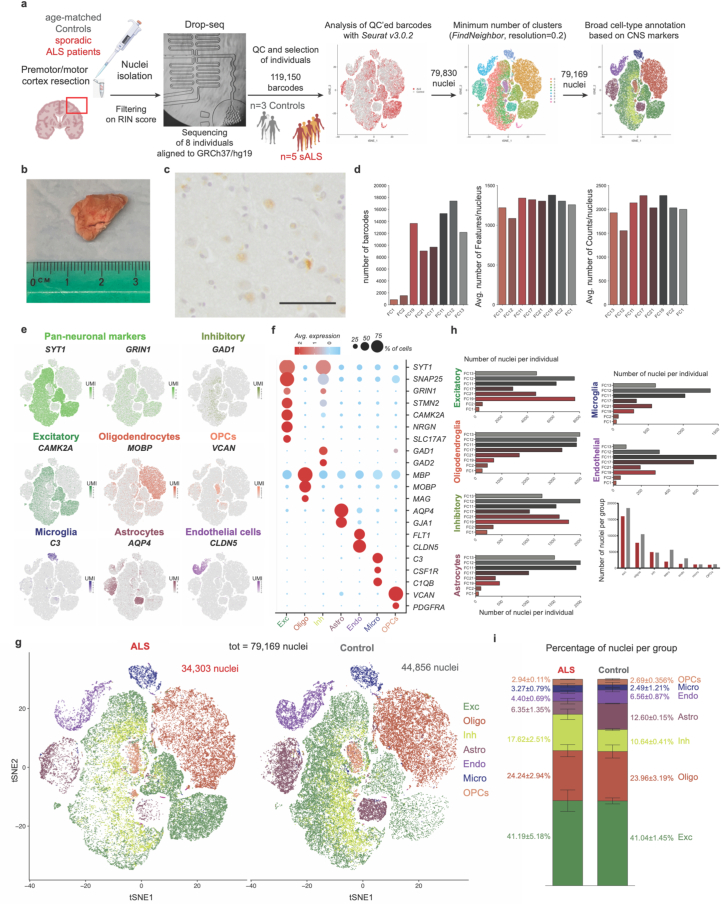
Technical parameters of snRNAseq and cell-type distribution across individuals. **a**. Schematic diagram of workflow for isolation of nuclei from cortices of ALS patients and age-matched controls followed by single-cell RNA sequencing by DropSeq, library generation and Quality Controls for analysis with Seurat 3.0.2 **b**. Frozen tissue from one of the individuals. **c**. Staining for TDP-43 in one of the patient sample, note neuron with skein-like inclusions and faint nuclear staining (scale bar 25*μ*m, n = 3 patients analysed). **d**. Quality controls post-filtering (FC – Frontal Cortex): number of total nuclei detected (barcodes), average number of genes per nucleus (nFeatures), and average number of UMIs (Unique Molecular Identifiers) per nucleus (nCounts). **e**. *t*-SNE projections of the whole cohort with expression of broad cell type markers. **f**. Dotplot representing percentage of cells expressing additional cell type specific markers. **g**. *t*-SNE distribution of whole cohort with annotated cell types split by diagnosis (ALS patients *n* = 5, age-matched Controls *n* = 3, *n* = 79,169 total nuclei). **h**. Quality controls post-filtering (FC – Frontal Cortex): number of total nuclei detected (barcodes), average number of genes per nucleus (nFeatures), and average number of UMIs (Unique Molecular Identifiers) per nucleus (nCounts). **i**. Fraction of each cell types identified in whole cohort split by diagnosis (mean ± SEM, ALS patients *n* = 5, age-matched Controls *n* = 3).

We used snRNAseq to profile motor/premotor cortical gray matter from patients with sALS and age-matched controls with no neurological disease using Drop-seq technology^14^ (Fig. 1a, Source Data Table 1 and Extended Data Fig. 1a–c). After screening for RNA quality, 79,169 barcoded droplets from eight individuals were analyzed (*n* = 5 sALS and *n* = 3 control), with a mean of 1,269 genes and 2,026 unique molecular identifiers (UMIs) (Extended Data Fig. 1d). We used Seurat^15^, the single-cell analysis package, to cluster and annotate groups according to canonical markers of brain cell types^16^: excitatory and inhibitory neurons, oligodendrocytes, oligodendrocyte progenitor cells (OPCs), microglia, astrocytes and endothelial cells (Extended Data Fig. 1e,f). The observed cell type distribution corresponded to previous studies^16^ and enabled robust categorization for downstream analysis. Cellular distribution was homogeneous between sexes and individuals, except for a modestly lower number of astrocytes in ALS samples (Extended Data Fig. 1g–i).

### Elevated expression of ALS–FTD genes in a specific class of neurons

**Extended Data Fig. 2:**
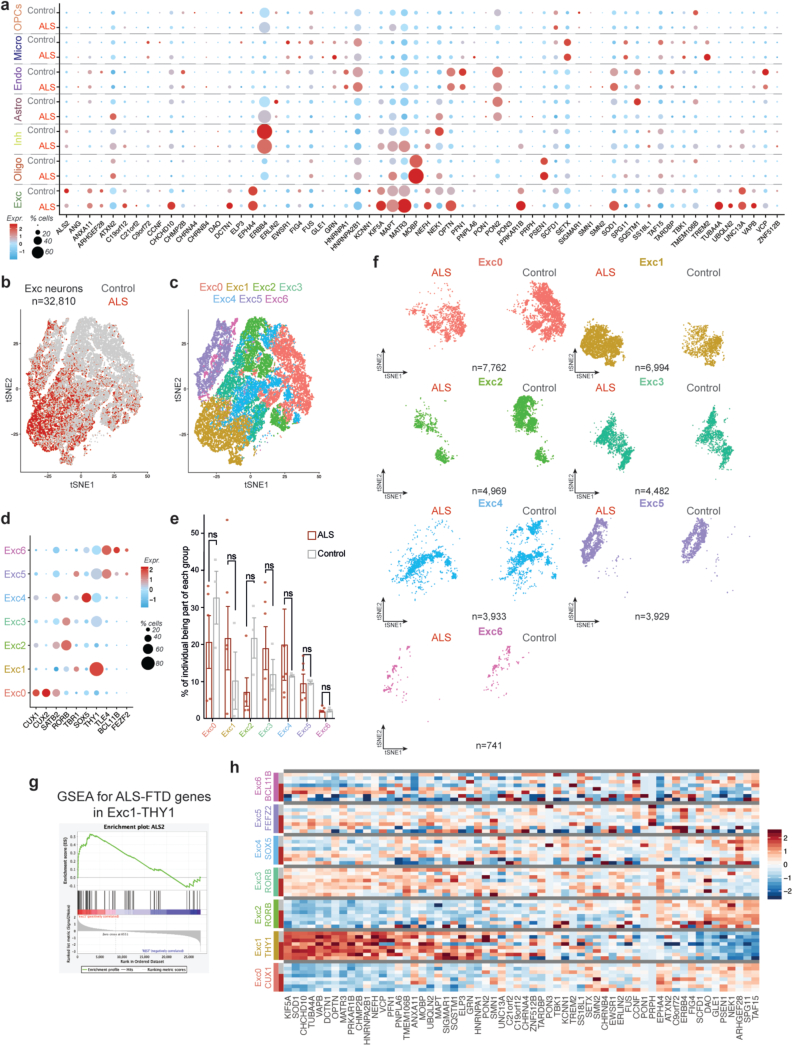
Expression of ALS-FTD associated genes in different cellular subtypes and excitatory neurons subtypes. **a**. Dotplot representing expression of gene associated with the ALS-FTD spectrum in each cell type identified in the whole cortex split by diagnosis. **b**. *t*-SNE projection of excitatory neurons clusters (ALS *n* = 15,227 nuclei, Control *n* = 17,583 nuclei). **c**. *t*-SNE projection of subclusters identified in excitatory neurons represents different, biologically relevant neuronal layers (*FindNeighbor*(res=0.2)). **d**. Dotplot representing percentage of cells expressing broad markers for different cortical layers. **e**. Distribution of excitatory neurons in subclusters by diagnosis (mean ± SEM). **f**. *t*-SNE projection of excitatory neurons by clusters and by diagnosis. **g**. Gene Set Enrichment Analysis for the ALS-FTD associated genes in Exc1 excitatory neuron subtype. **h**. Heatmap representing expression of gene associated with the ALS-FTD spectrum in each excitatory neurons identified split by diagnosis.

To potentially identify cell types underlying ALS pathophysiology, we examined the expression of known familial genes for ALS–FTD and variants identified as risk factors from genome-wide association studies (GWAS). These genes were expressed to a variable degree between cell types and many of them were ubiquitously expressed as already known^2,7^ (Extended Data Fig. 2a). We then computed a ‘module score’ for this set of genes^17^; this metric generates a standardized *z*-score for the expression of each gene and sums it up as a total score for the gene set. A positive score suggests higher expression of the gene set compared with the average expression of the module across the dataset. We computed parallel module scores for lists from the latest GWAS for disorders that affect the cortex but not specifically Betz cells: Alzheimer’s disease (AD)^18,19^ and multiple sclerosis (MS)^20^ (Fig. 1a and Source Data Table 2). No clear enrichment for the ALS–FTD gene list was identified (Fig. 1b), as anticipated by the scattered, ubiquitous expression pattern. On the other hand, AD and MS modules showed respective enrichment in microglia, as expected on the basis of the strong immune signature in these diseases^18–20^ (Fig. 1c,d). These results corroborate knowledge in the field, underlying the strength of this analysis, and confirm our results in an unbiased, single-cell resolution.

Considering the selective loss of neurons in ALS^2^, we further analyzed these cells. We found 32,810 nuclei from excitatory neurons with unbiased clustering identifying seven subgroups (Exc0–6) expressing known markers of different cortical layers^16^ (Extended Data Fig. 2b–f). Analysis of the ALS–FTD module in these cells showed a positive score in *THY1*-expressing subgroup Exc1 (normalized enrichment score of 1.834) (Fig. 1e and Extended Data Fig. 2g,h) and no significant enrichment for AD and MS modules (Fig. 1f,g). We decided to investigate the identity of these cells and the possibility of them being ETNs.

**Fig. 2:**
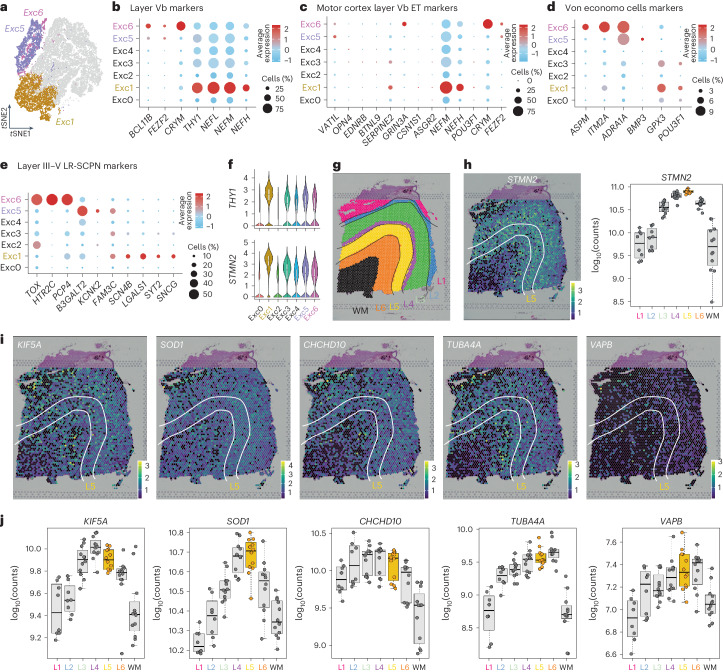
ALS–FTD susceptible neurons are layer V ETNs. **a**, *t*SNE projection of presumptive layer V neurons. **b**, A dot plot representing expression of layer V markers **c**, A dot plot for markers of LVb ETNs of human motor cortex. **d**, A dot plot representing expression of von Economo markers. **e**, A dot plot representing expression LR-SCPN markers. **f**, Representative violin plot for markers of layer V ETNs of human motor cortex (geometric box plots for median and interquantile ranges). **g**, Visual depiction of layers identified by Maynard et al. 2021 (*n* = 7, **g**–**j**, publicly available) (L, layer; WM, white matter). **h**, A spot plot depicting expression of layer Vb motor cortex marker, *STMN2*, identified as enriched in THY1-Exc1, with corresponding box plot quantification. **i**,**j**, Box plots (**j**) and corresponding spot plots (**i**) for the expression of the top five ALS–FTD-associated genes expressed in Exc1 (box plots for mean and interquantile ranges). Source data

**Extended Data Fig. 3:**
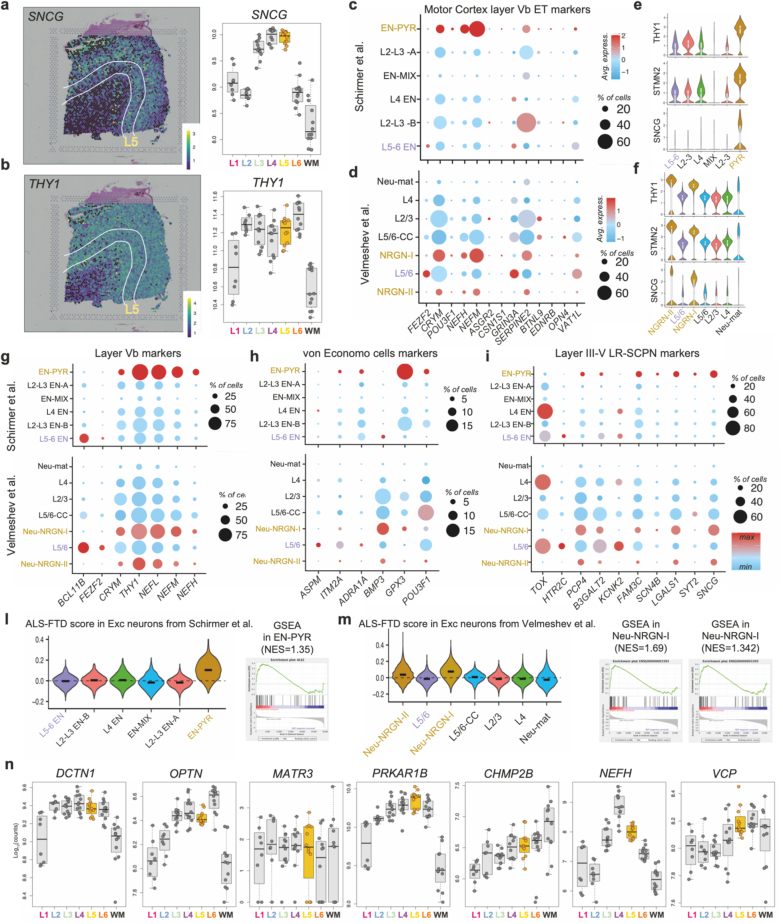
L5-ETNs/CSMNs-like neurons express higher levels of ALS-FTD related genes. **a,b**, Spotplot and corresponding boxplot from Maynard et al. for the expression of layer Vb Motor Cortex marker, *SNCG* and *THY1*, identified as enriched in Exc1 (boxplots for mean and interquantile ranges + -SD). **c,d**. Dotplot and representative Violin plots for markers of L5 ExtraTelencephalic neurons of human Motor Cortex in Schirmer et al. **e,f**. Dotplot and representative Violin plots for markers of L5 ExtraTelencephalic neurons of human Motor Cortex in Velmeshev et al. (geometric boxplots for median and interquantile ranges) **g-i**. Dotplot representing expression of Layer V markers (d), von Economo markers (e), LR-SCPN markers (f) in Schirmer et al. and Velmeshev et al. **l**. Violin plots and corresponding Gene Set Enrichment Analysis of z-scores for expression of ALS-FTD-associated genes in THY1-neurons identified by Schimer et al. (bars denote median). **m**. Violin plots and corresponding Gene Set Enrichment Analysis of z-scores for expression of ALS-FTD-associated genes in THY1-neurons identified by Velmeshev et al. (bars denote median). **n**. Boxplot from Maynard et al. for the expression of additional top ALS/FTD associated genes identified in Exc1 (geometric boxplots for mean and interquantile ranges + -SD) *(n=multiple individuals as reported in publicly available datasets)*

We identified three subgroups expressing markers of subcerebral projection neurons: Exc1, Exc5 and Exc6 (Fig. 2a). Exc5 and Exc6 expressed canonical genes *FEZF2*, *BCL11B* and *CRYM*^21^; Exc1 expressed *THY1*, enriched in human layer V^22^ and used as a reporter for CSMNs^23^, and high levels of neurofilament chains, markers of ETNs^24^ (Fig. 2b). Recent reports dissected the transcriptomic identity of layer V extratelencephalic neurons in the human motor cortex^24^. We detected ETNs markers in these groups, with Exc1 expressing *SERPINE2* and *POU3F1*, specific of ETNs^24^, and *NEFH* and *STMN2*, broad markers of motor neurons (MNs)^25^ (Fig. 2c). Owing to the anatomical location of our samples and the presence of ETNs across motor-related areas, we plotted genes specific to layer V ETNs von Economo cells^26,27^, affected in FTD and other long-range subcerebral projecting neurons (LR-SCPNs)^28^ and confirmed that all three groups expressed these markers (Fig. 2d,e). To further characterize these patterns, we leveraged a publicly available single-cell, spatial dataset of control human dorsal cortex^29^. We confirmed that markers expressed in Exc1 *THY1*, *STMN2* and *SNCG* (Fig. 2f) are specifically expressed in layer V (L5) (Fig. 2g,h and Extended Data Fig. 3a,b). This evidence suggests that Exc1, Exc5 and Exc6 express markers of layer V extratelencephalic neurons of cortical areas affected by ALS–FTD.

To further confirm that *THY1*^high^ neurons expressed higher levels of ALS–FTD genes, we ran a module score analysis in two datasets that identified *THY1*^high^ excitatory neurons^22,30^. In these studies, *THY1* neurons expressed ETN markers, layer V, von Economo and LR-SCPN markers (Extended Data Fig. 3c–i) and expressed higher levels of the ALS–FTD module (Extended Data Fig. 3l–m). Analysis of the spatial transcriptomic dataset^29^ confirmed that the top ten ALS–FTD-associated genes most highly expressed in Exc1 (Extended Data Fig. 2g) are expressed in deeper layers of the cortex and specifically in layer V (Fig. 2i,j and Extended Data Fig. 3n). Larger cohorts of patients and validations at the protein level are needed to confirm the degree of dependency of ETNs on this gene set but studies in human^31^ and mouse^32^ showed that deep-layer neurons have a higher propensity to form TDP-43 aggregates, the hallmark of ALS–FTD. Here, we provide a possible link to their specific vulnerability.

### Higher cellular burden in deeper-layer excitatory neurons

**Fig. 3:**
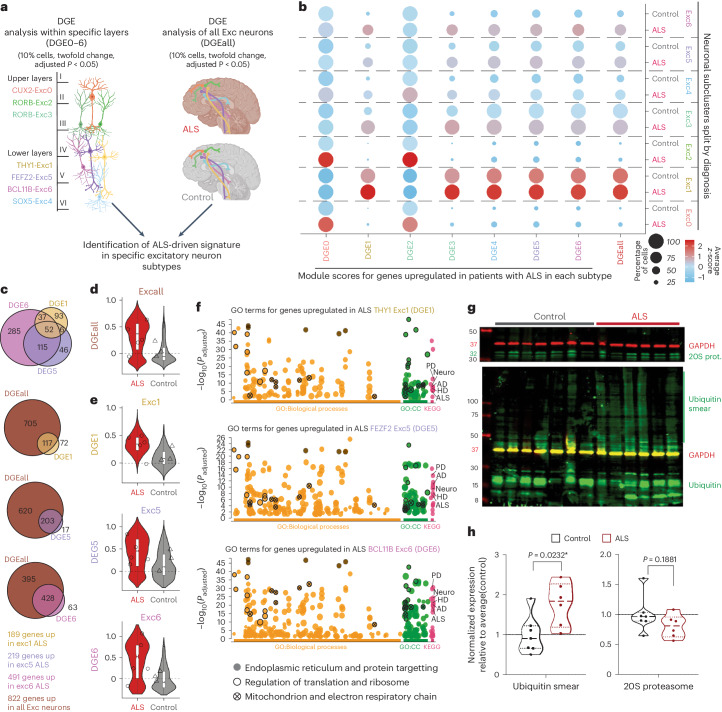
ALS excitatory neurons present increased expression of stress-related pathways. **a**, A schematic of DGE analysis. **b**, A dot plot representing scores for genes upregulated in each subgroup of Exc neurons (DGE0–6) and globally upregulated in all Exc (DGEall). **c**, Comparison of genes globally upregulated in ALS (DGEall) with genes upregulated in classes of L5-ETNs (genes expressed by >10% of cells, >2 FC and adjusted *P* value <0.05). **d**, Violin plots of *z*-scores for genes globally upregulated in all excitatory cells (DGEall) in all excitatory neurons (*n* = 3 controls, *n* = 5 patients with sALS; geometric box plots represent median and interquantile ranges and symbols indicate average score per individual). **e**, Violin plots of *z*-scores for genes upregulated in classes of L5 ETNs (DGE1, DGE5 and DGE6) in the three groups (geometric box plots represent median and interquantile ranges, symbols indicate average score per individual). **f**, GO analysis for genes upregulated in L5 ETNs classes (DGE1, DGE5 and DGE6); the highlighted terms are shared between the three. PD, Parkinson’s Disease; AD, Alzheimer’s Disease; HD, Huntington’s disease; CC, cellular components; KEGG, Kyoto Encyclopedia of Genes and Genomes. **g**–**h**, Western blot quantification of ubiquitin accumulation and 20S proteasome (prot.) subunit from motor cortices of separate cohort of patients with ALS (*n* = 6) and age-matched controls (*n* = 7) (*t*-test).

We next examined whether the enriched expression of ALS–FTD genes might relate to changes that occur in excitatory neurons in response to ALS. We conducted differential gene expression (DGE) analysis between neurons from patients and controls, across all excitatory cells and within each subgroup (Fig. 3a). We selected genes significantly upregulated in patients globally (DGEall) and within each subgroup (DGE0–6), calculated module scores for each set and investigated whether certain neuronal subtypes might have similar responses to ALS (Source Data Table 3). We found a correlation between scores in groups expressing deep-layer markers and the global changes identified in patients (Fig. 3b), suggesting that pathology in lower cortical layers might be driving observed alterations. For instance, groups expressing ETN markers (Exc1, Exc5 and Exc6) shared many upregulated genes with each other and with the global signature (Fig. 3c). Intriguingly, this class of genes is, like genetic risk factors, constitutively expressed at higher levels in Exc1 ETNs (Fig. 3b), advocating for a proposed interplay between genetics and molecular pathways that sensitizes ETNs to ALS^33^.

**Extended Data Fig. 4:**
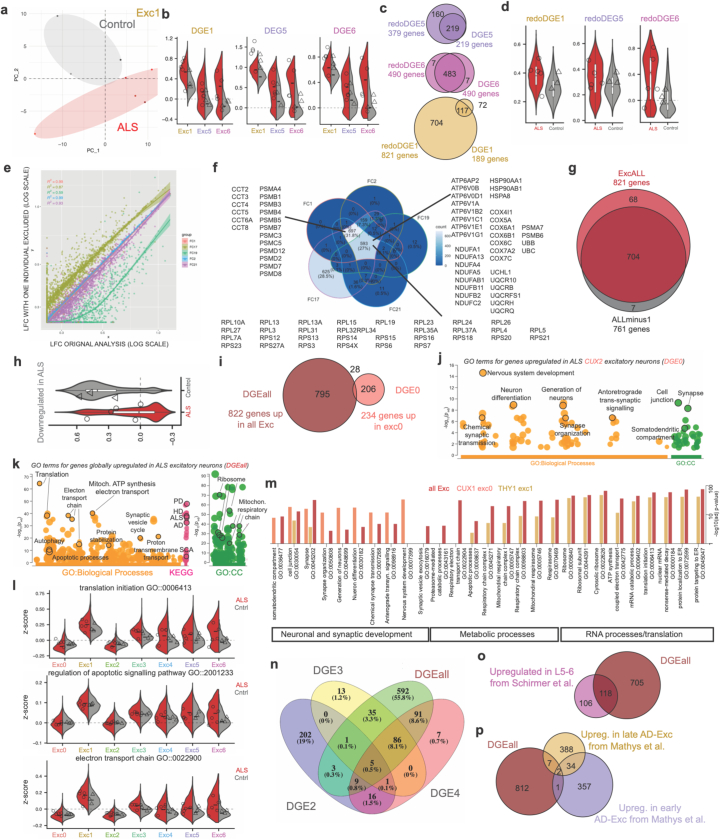
Classes of L5-ETNs express higher levels of stress pathways. **a**. DGE-based PCA plots showing separation of individuals by diagnosis in Exc1. **b**. Violin plots of z-scores for genes upregulated in each class of L5-ETNs (DGE1, DGE5, DGE6) in the three groups split by diagnosis (bars denote median – symbols: average score per individual). **c**, Comparison of DGEs signature (>10% cells, >2-FC, adj.p < 0.05) and redoDGE signatures identified in each subgroup with random seeding of equal cell numbers per diagnosis (>10% cells, >2-FC, adj.p < 0.05, max.cells.per.ident=lowest.number.per.group). **d**. Violin plots of z-scores for redoDGE signatures (median and interquantile ranges – symbols: average score per individual). **e**. Correlation plot of Log Fold Changes in ALLminus1 comparisons. **f**. Venn diagram comparing genes shared between ALLminus1 analyses. **g**. Venn diagram comparing genes in common between ALLminus1 analysis and ExcAll. **h**. Violin plots of z-scores for genes globally downregulated in Excall (geometric boxplots represent median and interquantile ranges – symbols: average score per individual). **i**. Comparison of genes globally upregulated in ALS (DGEall) with genes upregulated in CUX2-exc0 (>10% of cells, >2-FC, adj.p < 0.05) **j**. Gene Ontology analysis of terms for DGE0 highlighted terms involved in synaptic biology (CC=Cellular Components). **k.** Gene Ontology analysis of terms for DGEall highlighted terms involved in synaptic biology (CC=Cellular Components). **l**,**m.** Selected GO terms for DGE0, DGE1 and DGEall. **n.** Venn Diagram for shared upregulated genes between other excitatory neurons and global signature. **o**,**p.** Venn Diagrams for shared upregulated genes between treated MS excitatory neurons from Schirmer et al. and AD excitatory neurons from Mathys et al. with changes found in this study.

Owing to the small cohort size and because of the diverse etiology of ALS–FTD^6^, we decided to test how shared these changes were and test whether they were driven by a single individual. We started by averaging gene expression by sample within groups of ETNs and ran principal component analysis (PCA) on the individual-aggregated matrices that showed widespread diversity between both patients and controls with partial segregation by diagnosis (Extended Data Fig. 4a). This could be explained by a compound effect of diversity of human population, sampling of different cells per individual or different disease etiology. To avoid differential gene expression analyses confounded by this effect, we carried out several preanalyses to corroborate the strength of our study and come to more confident conclusions.

We proceeded to compute module scores for the global sALS signature (DGEall) and sALS signatures in ETNs (DGE1,5,6) and saw that, even though with intragroup variability, these signatures showed higher scores in ALS at single-cell and aggregated expression level with similarities between groups of ETNs (Fig. 3d and Extended Data Fig. 4b). To ensure that these signatures were driven by disease changes, we recalculated differentially expressed genes (DEGs) by down-sampling each group of ETNs to the smallest number of cells by diagnosis (that is, redoDGE1,5,6). This analysis confirmed the great overlap of DGE signatures and redoDGE signatures and that these signatures were higher in sALS at single-cell resolution and average level (Extended Data Fig. 4c,d).

Finally, to further test whether the results were driven by the transcriptome of a single individual, we repeated the DGE analysis by excluding cells from one patient at a time. This analysis shows that, even though different subsets of genes are detected in each analysis, the shared ones show a similar direction and amplitude in changes (Extended Data Fig. 4e). This comparison shows that singular gene changes might be sporadically driven by one individual and because of the small cohort size, it would be hard to discern between biological or technical outliers. However, the shared DEGs (>65% in this case; Extended Data Fig. 4f) and the general direction of the ALS-driven signature are maintained, with genes commonly unregulated in at least four out of five patients (ALLminus1 list) having >85% overlap with DGEall (Extended Data Fig. 4g). We proceeded to use only genes shared by at least four individuals for subsequent analyses for all cell types.

**Extended Data Fig. 5:**
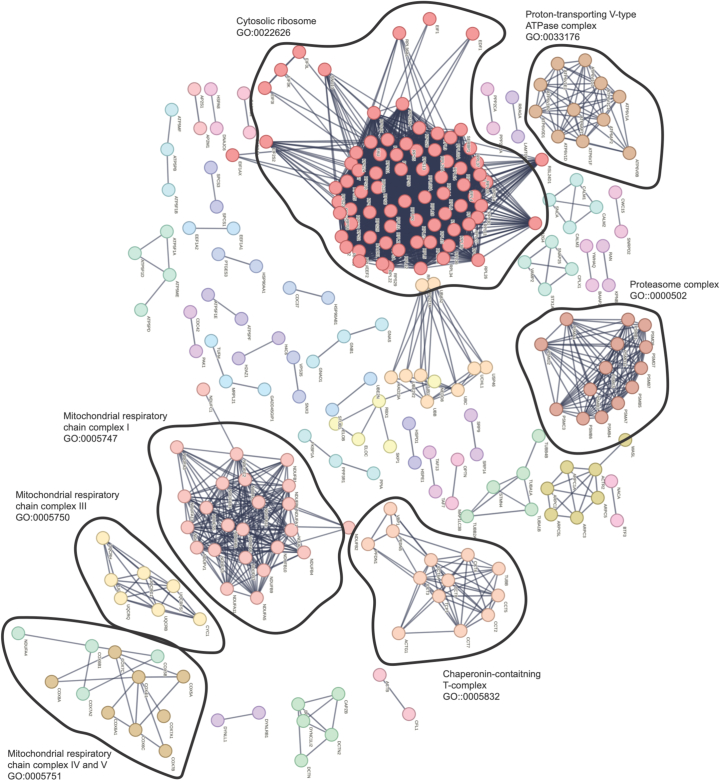
Global protein-protein interaction network for genes upregulated in ALS excitatory neurons. Color-coding derived from MCL clustering to identified closely related groups of proteins.

We ran Gene Ontology (GO) analysis and showed that DEGs identified in classes of ETNs are connected to cellular stresses previously associated with ALS even from studies with hundreds of patients^7,34^ (Fig. 3f). Interactome analysis revealed coordinated alterations in genes that function in translational machinery, mitochondria, protein folding, proteostasis and degradation pathways connected to the proteasome and shared many transcriptional changes with patients’ excitatory cells as a whole (Extended Data Figs. 4k–m and 5). Interestingly, genes upregulated in upper layers of the cortex, a region relatively spared of pathology, shared less similarities with DGEall and were associated with synaptic biology (Extended Data Fig. 4h–j). Comparison of ALS-driven transcriptomic changes with other studies underlined similarities with genes upregulated in excitatory neurons from MS^22^ but not from AD patients^35^ (Extended Data Fig. 4n,o), suggesting that there are similar processes at the origin of neurodegeneration but that these changes are not universal. The analyses so far have highlighted the interindividual variability intrinsic of this kind of datasets and the particular attention studies need to divert into assuring reproducibility of the results. Nonetheless, we provide a snapshot of disruption in neuronal health in patients with ALS, in which lower layers of cortical excitatory neurons share higher levels of cellular stresses.

**Fig. 4:**
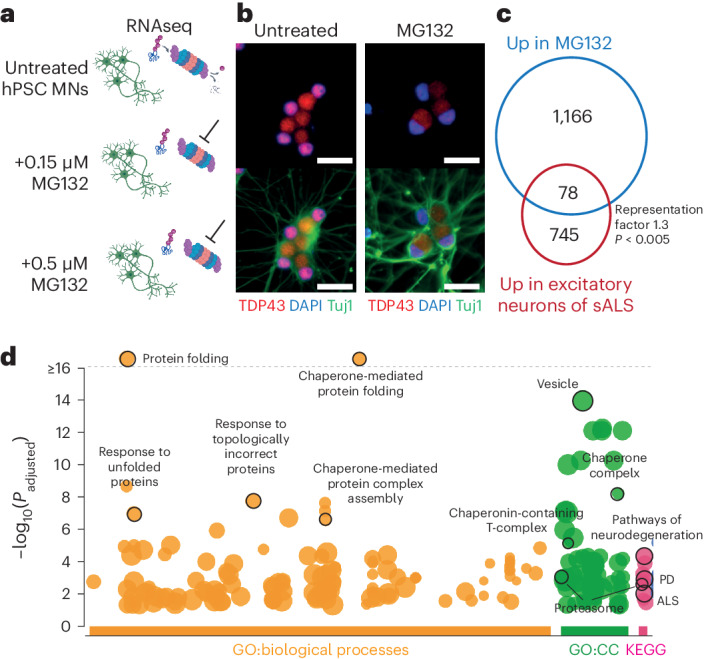
Proteostatic stress in hPS cell-derived neurons resembles changes in excitatory neurons from brain of patients with ALS. **a**, Diagram of neuronal differentiation from PSCs and treatment with proteasome inhibitors for bulk RNA sequencing. **b**, Immunofluorescence of TDP-43 localization after treatment. **c**, Venn diagram depicting shared upregulated genes between treated hPS cell-derived neurons and excitatory neurons from patients with ALS. **d**. GO analysis for shared genes in **c**, highlighted terms involved in protein folding and neurodegenerative diseases. CC, cellular components. Source data

**Extended Data Fig. 6:**
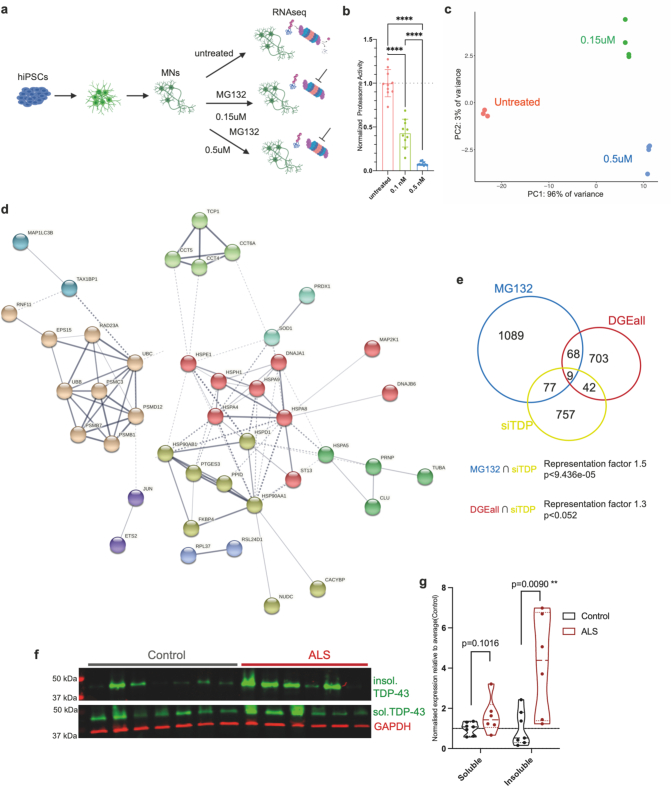
Proteostatic stress in hPSC-derived neurons resembles changes in excitatory neurons from brain of ALS patients. **a**. Diagram of neuronal differentiation from Pluripotent Stem Cells and treatment with proteasome inhibitors for bulk RNA-sequencing. **b**. Quantification of proteasome inhibition (mean ± SD, n = 3 biological replicates, n = 9 technical total, (t-test, ****p < 0.001). **c**. Principle Component Analysis plot showing strong effect of treatments compared to untreated controls (n = 3 biological replicates). **d**. Protein-protein interaction network of shared genes. **e**. Venn Diagram depicting shared upregulated genes in treated hPSC-derived neurons (MG132), excitatory neurons from ALS patients (DGEall) and genes misregulated in human neurons after TDP-43 siRNA from Klim et al. (proportional test) **f-g**. Western Blot quantification of soluble and insoluble TDP-43 from motor cortex of ALS patients and age-matched controls (t-test). Source data

Next, we sought to determine what proportion of this transcriptomic signature may be associated with proteostatic stress specifically in neuronal cells. Presently, in vitro modeling of sporadic ALS requires high numbers of lines, high-throughput methods and needs further standardization^3,36^. To overcome these limitations, we implemented transient proteasome inhibition as a highly reproducible, dose-responsive, temporally controlled model to induce TDP-43 nuclear loss as seen in patients’ Betz cells and other ALS-related dysfunction in human neurons^4,37,38^ (Fig. 4a and Extended Data Fig. 6a,b). Application of a proteasome inhibitor to human pluripotent stem (hPS) cell-derived neurons^37^ induced nuclear loss of TDP-43 (Fig. 4b). Bulk RNA sequencing analysis showed widespread changes after treatment, with a significant overlap of upregulated genes between stressed hPS cell-derived neurons and sALS neurons (Fig. 4c), specifically proteasome subunits and heat-shock response-associated chaperonins, confirmed by GO analysis of shared genes (Fig. 4d and Extended Data Fig. 6d). Moreover, genes upregulated after inhibition show a significant overlap with transcripts misregulated after downregulation of TDP-43 in neurons^37^ (Extended Data Fig. 6e). This confirms that some changes identified in sALS neurons are connected to neuronally intrinsic proteostasis and are at least in part connected to alterations in TDP-43.

To confirm hindered proteostasis in ALS cortex, we selected a second cohort of patients with sALS and controls for biochemical evaluation. We extracted protein, confirmed increased insoluble TDP-43 in patients (Extended Data Fig. 6f,g) and showed that, despite the presence of core proteosomal subunits, pathology is accompanied by the accumulation of highly ubiquitinated proteins (Fig. 3g,h), the hallmark of impaired proteostasis. These findings suggest that the neuronal stress observed in ETNs from patients with ALS represents an intrinsic susceptibility to proteostatic stress orchestrated, in part, by abnormal homeostasis of TDP-43.

### Oligodendroglia respond with a neuronally engaged state

**Fig. 5:**
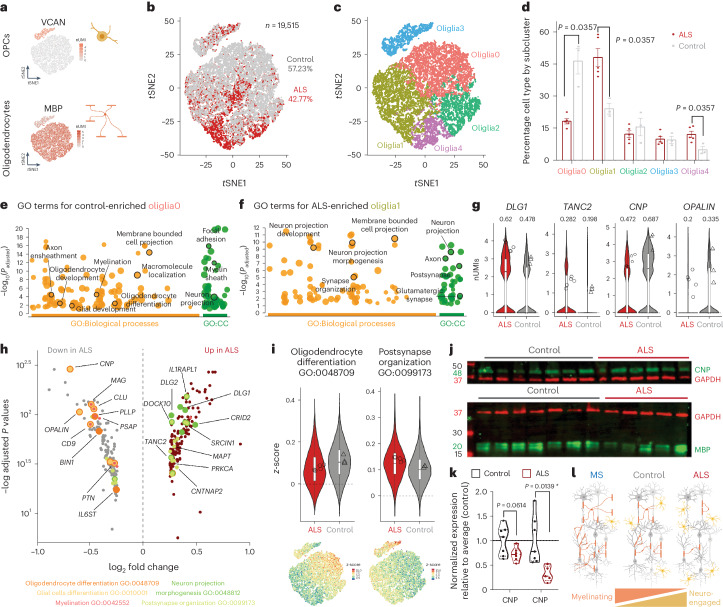
In ALS, oligodendroglial cells decrease their myelinating machinery in favor of a neuro-engaged state. **a**, *t*SNE projection of OPCs and oligodendrocytes markers. **b**, *t*SNE projection of oligodendroglia (ALS *n* = 8,372 nuclei and control *n* = 11,168 nuclei). **c**, *t*SNE projection of subclusters within oligodendroglia (Wilcoxon–Mann–Whitney). **d**, Distribution of subclusters by diagnosis) (mean ± s.e.m. **e**, GO analysis for genes characteristic of control-enriched oliglia0 highlighted terms involved in myelination. CC, cellular components. **f**, GO analysis for genes characteristic of ALS-enriched oliglia1 highlighted terms involved in neuro-engaged functions. **g**, Violin plots of representative genes for neuro-supportive functions (left) and myelination (right) (geometric box plots for median and interquantile ranges; symbols indicate log_2_(average expression) per individual (fraction of cell expressing). **h**, Volcano plot of DEGs in oligodendroglia. Highlighted genes identified in GO terms related to myelination (orange) and neuro-engaged functions (green). **i**, Violin plots representing *z*-score for selected GO terms and related *t*SNE projection (boxplot representing median and interquantile ranges; symbols indicate average score per individual). **j**,**k**, Western blot (**j**) and quantification (**k**) of CNPase and MBP from motor cortices of patients with ALS and age-matched controls (*t*-test). **l**. Diagram illustrates shift of oligodendrocytes states (*t*-test) (for **a**–**l**, *n* = 3 control and *n* = 5 patients with sALS). nUMI - normalized Unique Molecular Identifier.

**Extended Data Fig. 7:**
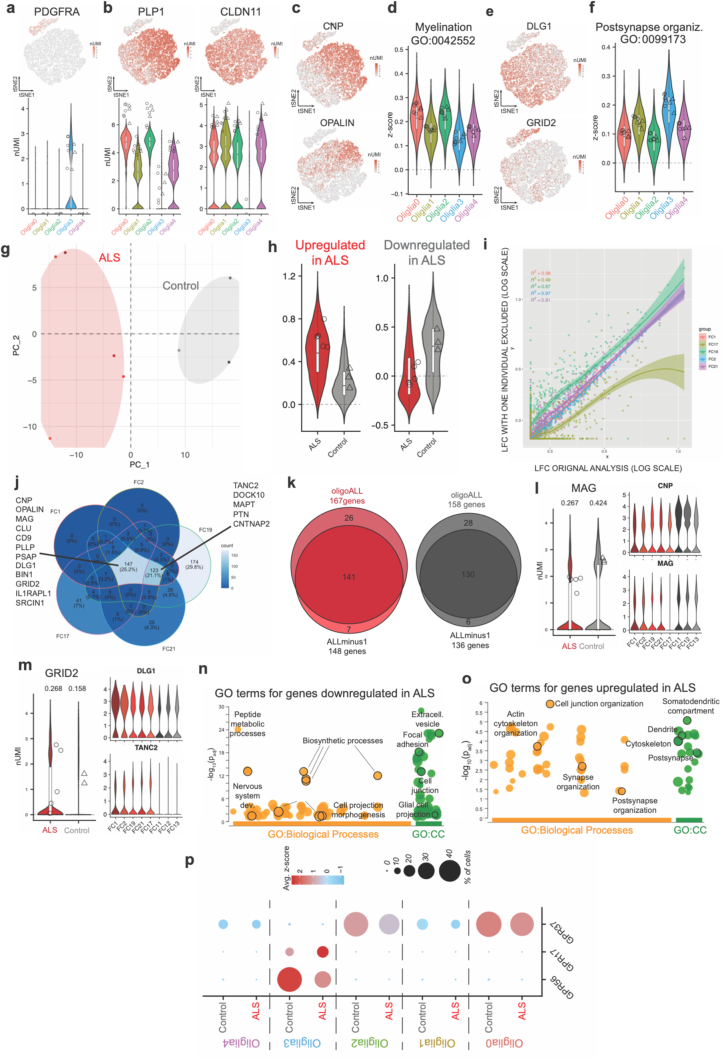
Oligodendrocytes polarize between myelinating and neuro-engaged states. **a,b**. *t*-SNE projection and Violin plot of markers of Oligodendrocyte Progenitor Cells (OPCs) and mature oligodendrocytes (geometric boxplots represent median and interquantile ranges – symbols: log2(AverageExpression) per individual) (fraction of cell expressing). **c,d**. *t*-SNE projection of markers of actively myelinating oligodendrocytes and violin plots representing z-score for selected GO terms by cluster (geometric boxplots represent median and interquantile ranges – symbols: average score per individual). **e,f**. *t*-SNE projection of markers of neuronally-engaged oligodendrocytes and violin plots representing z-score for selected GO terms by cluster (geometric boxplots represent median and interquantile ranges – symbols: average score per individual). **g**. DGE-based PCA plot showing separation of individuals by diagnosis. **h**. Violin plot for z-score for genes upregulated and downregulated in ALS patients (geometric boxplots represent median and interquantile ranges – symbols: average score per individual). **i**. Correlation plot of Log Fold Changes in ALLminus1 comparisons. **j**. Venn diagram comparing genes shared between ALLminus1 analyses. **k**. Venn diagram comparing genes in common between ALLminus1 analysis and OligoAll. **l**. Violin plot of markers of actively myelinating oligodendrocytes (geometric boxplots represent median and interquantile ranges – symbols: log2(AverageExpression) per individual) (fraction of cell expressing). **m**. Violin plot of markers of neuronally-engaged oligodendrocytes (geometric boxplots represent median and interquantile ranges – symbols: log2(AverageExpression) per individual) (fraction of cell expressing). **n**,**o**. GO analysis for genes downregulated and upregulated in ALS oligodendrocytes, highlighted terms involved in myelination (CC=Cellular Component). **p**. Dotplot representing genes characteristic of maturation and development of OPCs in myelinating oligodendrocytes in each subcluster split by diagnosis.

To extend deep into the cord, ETNs are dependent on robust axonal integrity^39^. As others detected changes in myelination in ALS^12^ and in FTD^40^, we analyzed 19,151 nuclei from myelinating cells that clustered into five groups: one of OPCs—oliglia3, and four of oligodendrocytes—oliglia0, 1, 2, 4 (Fig. 5a–c and Extended Data Fig. 7a,b). We noted a significant shift of ALS nuclei representation in oliglia0 versus oliglia1 and oliglia4 (Fig. 5d). Control-enriched oliglia0 were characterized by GO terms connected to oligodendrocyte development and myelination and expressed higher levels of myelinating genes, for example, *CNP*, *OPALIN* and *MAG* (Fig. 5e and Extended Data Fig. 7c,d). ALS-enriched oliglia1 showed terms for neurite morphogenesis, synaptic organization and higher expression of synaptic-related genes *DLG1*, *DLG2* and *GRID2* (Fig. 5f and Extended Data Fig. 7e,f). Intriguingly, expression of neuronal enriched transcripts has been found in oligodendrocytes in primate motor cortex^24^.

While intragroup variability was apparent between aggregated oligodendroglial transcriptomic signatures, there was a demarcated separation of individuals by diagnosis with global gene expression conserved at single-cell and aggregated levels (Extended Data Fig. 7g,h). We proceeded to calculate DEGs by excluding one individual at a time (ALLminus1 approach described above), selected genes commonly unregulated in at least four out of five patients and compiled gene lists for subsequent analyses (Extended Data Fig. 7i–k). Altogether, DGE and GO analyses support a shift of sALS oligodendrocytes from myelinating to neuronally engaged states with upregulation of genes involved in synapse modulation and decrease of regulators of myelination (Fig. 5g–i and Extended Data Fig. 7l–o). Loss of myelination is exemplified by the changes in G-protein coupled receptors (GPRCs) marking developmental milestones: *GPR56*, expressed in OPCs^41^, and *GPR37*, expressed in myelinating cells^42^, were lowly expressed in ALS-enriched groups and globally downregulated (Extended Data Fig. 7p).

**Extended Data Fig. 8:**
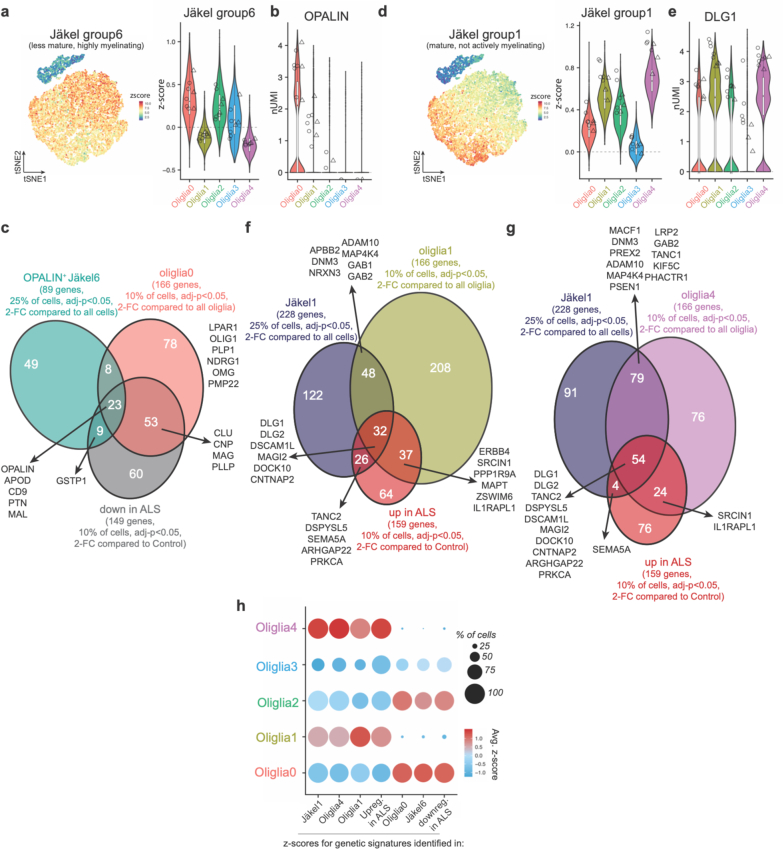
Comparison of ALS-driven changes with study with similar signature disrupted in disease (MS). **a,b**. *t*-SNE projection and violin plot representing z-score for highly myelinating, OPALIN^+^ oligodendrocytes in Jäkel et al (geometric boxplots represent median and interquantile ranges – symbols: average per individual). **c**. Comparison of genes downregulated in oligodendroglia from ALS patients with genes characteristic of highly myelinating, OPALIN^+^ subtypes identified by this study (oliglia0) and by Jäkel et al (Jäkel6), highlighted genes are shared with GO terms shown in figures. **d,e**. *t*-SNE projection and violin plot representing z-score for genes of mature, not-actively myelinating oligodendrocytes in Jäkel et al (geometric boxplots represent median and interquantile ranges – symbols: average per individual). **f,g**. Comparison of genes upregulated in oligodendroglia from ALS patients with genes characteristic of mature, lowly myelinating groups in this study (oliglia1 and 4) and by Jäkel et al (Jäkel1), highlighted genes are shared with GO terms shown in figures. **h**. Dotplot representing z-scores for the genetic signatures identified in the actively myelinating cells, the mature lowly myelinating cells and DEGs identified in this study. *(for whole panel n* = *3 Control individuals, n* = *5 sALS patients)*

To further explore these changes, we compared them to published reports that identified shifts in oligodendrocytes in MS (Source Data Table 4)^43^. Comparison of Jäkel et al.^43^ with our study revealed that control-related oliglia0 most closely resembled highly myelinating *OPALIN*^*+*^ cells from Jäkel6 (Extended Data Fig. 8a–c), while ALS-associated oliglia1 and oliglia4 aligned to not-actively myelinating Jäkel1 (Extended Data Fig. 8d–h). To confirm this shift, we ran validations on protein extracts from motor cortices and showed that oligodendrocyte-specific, myelin-associated proteins CNP and MBP are downregulated in patients (Fig. 5j–k), consistent with studies identifying demyelination in patients with sALS^12^ and with bulk RNA sequencing studies that identified a decrease in myelinating markers^7^. The data so far show how activation of stress pathways in deep-layer neurons is juxtaposed to a shift in oligodendrocytes from active myelination to oligo-to-neuron contact (Fig. 5l).

### Microglia activate an endolysosomal response

**Fig. 6:**
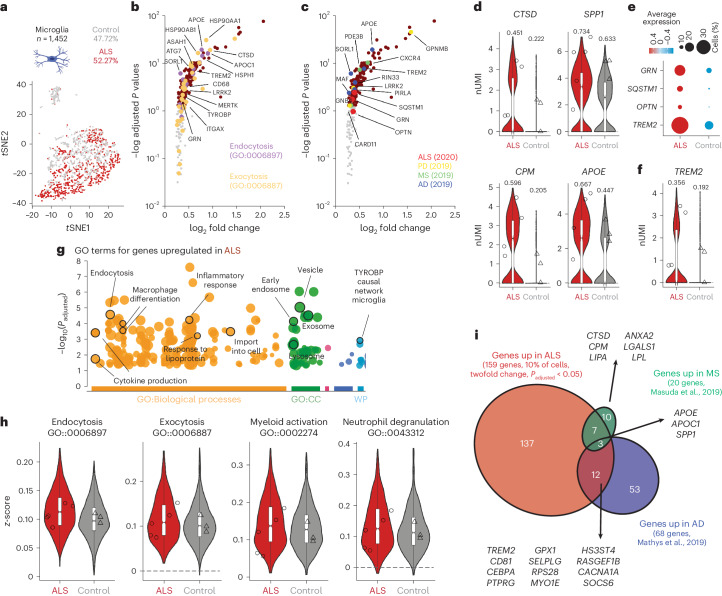
DAM signature in ALS. **a**, *t*SNE projection of microglia (ALS *n* = 759 nuclei and control *n* = 693 nuclei). **b**,**c**, Volcano plot of genes upregulated in microglia from ALS. Genes identified in GO terms for endocytosis and exocytosis (**b**) and genes associated with neurodegenerative diseases (**c**). **d**, Violin plots of representative genes upregulated in patients with ALS associated with reactive microglia (geometric box plots represent median and interquantile ranges; symbols indicate log_2_(average expression) per individual) (fraction of cell expressing). **e**, A dot plot representing expression of genes associated with ALS–FTD pathogenesis. **f**, Violin plots of representative ALS–FTD genes upregulated in ALS (geometric box plots represent median and interquantile ranges; symbols indicate log_2_(average expression) per individual) (fraction of cell expressing). **g**, GO analysis for genes upregulated in ALS microglia, highlighted terms involved in myeloid cells biology and/or pathogenesis of ALS (WP, WikiPathways). **h**, Violin plots representing *z*-scores for selected, statistically significant GO terms from **f** (geometric box plots represent median and interquantile ranges; symbols indicate average score per individual). **i**, Comparison of genes upregulated in microglia from ALS with genes upregulated in microglia in other neurodegenerative diseases (for **a**–**i**, *n* = 3 control individuals and *n* = 5 patients with sALS). nUMI, normalized Unique Molecular Identifier.

**Extended Data Fig. 9:**
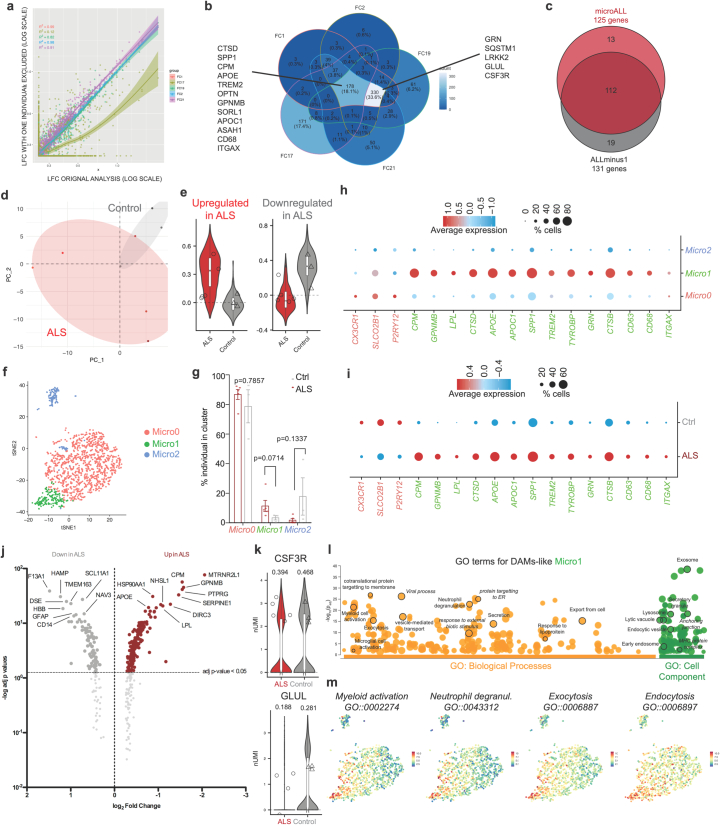
Shared features between ALS-driven changes and reactive subcluster of microglia. **a**. Correlation plot of Log Fold Changes in ALLminus1 comparisons. **b**. Venn diagram comparing genes shared between ALLminus1 analyses. **c**. Venn diagram comparing genes in common between ALLminus1 analysis and MicroAll. **d**. DGE-based PCA plot showing separation of individuals by diagnosis. **e**. Violin plot for z-score for genes upregulated and downregulated in ALS patients (geometric boxplots represent median and interquantile ranges – symbols: average score per individual). **f**. t-SNE projection of subclusters identified within microglia (Micro0 = Homeo = homeostatic, Micro1 = DAMs = Disease-associated microglia, Micro2 = Cycling cells)). **g**. Distribution of microglia within clusters by diagnosis. **h**. Dotplot representing genes identified as characteristic of Homeostatic microglia and DAMs by subcluster. **i**. Dotplot representing genes identified as characteristic of Homeostatic microglia and DAMs by diagnosis. **j**. Volcano plot of statistically significant differentially expressed genes between Control and ALS microglia (top ten upregulated and top ten downregulated genes highlighted). **k**. Violin plots of representative DEGs downregulated in ALS patients of genes associated with homeostatic microglia (geometric boxplots represent median and interquantile ranges – symbols: log2(AverageExpression) per individual) (fraction of cell expressing). **l**. Gene Ontology analysis of terms associated with genes characteristic of DAMs microglia, highlighted terms playing important role in microglial biology and/or pathogenesis of the disease. **m**. t-SNE projections representing z-score for selected, statistically significant GO terms.

Mouse models^44^, patient samples^5^ and function of ALS-related genes in myeloid cells^45^ have demonstrated the importance of microglia as modifiers of disease. In the 1,452 nuclei from microglia (Fig. 6a), comparative DGE analyses showed intragroup variability in magnitude but conserved directionality of changes and robustness of the core common biology (Extended Data Fig. 9a–e). We identified 159 genes upregulated in patients and, remarkably, many were associated with endocytosis and exocytosis, previously implicated in ALS^45^ (Fig. 6b,c). Several of these genes were also associated with microglial activation and neurodegenerative disorders (*CTSD, SPP1, CPM* and *APOE*) (Fig. 6c,d) and interestingly with ALS–FTD (*TREM2*, *OPTN*, *SQSTM1/p62* and *GRN*) (Fig. 5e,f). GO analysis for upregulated genes confirmed a proinflammatory state highlighting activation of endolysosomal pathways, secretion and immune cell degranulation previously associated with myeloid cells in ALS^45^ (Fig. 6g,h). Further subclustering identified three groups: homeostatic Micro0, ‘disease-associated microglia (DAM)’-like Micro1 and cycling Micro2 (Extended Data Fig. 9f–h). Notably, genes that characterized Micro1 were also upregulated in sALS (Extended Data Fig. 9i,j), in conjunction with a downregulation of homeostatic genes and upregulation of reactive pathways (Extended Data Fig. 9k–m).

**Extended Data Fig. 10:**
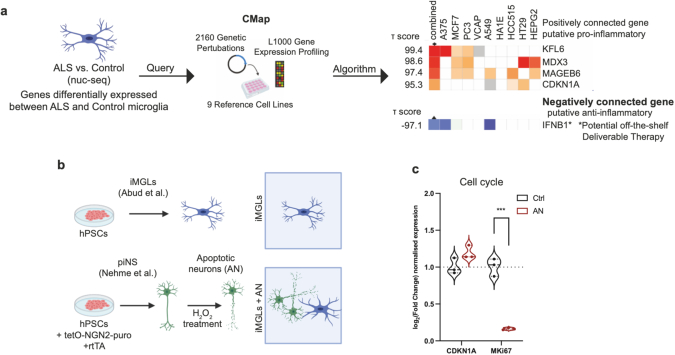
Apoptotic neurons upregulate lysosomal genes in microglia. **a**. Schematic of workflow and results from the Connectivity Map project for the genes upregulated in ALS microglia. Heatmap shows what cellular signature is most closely related to the query. **b**. Diagram of microglia and neuronal differentiation from Pluripotent Stem Cells, induction of apoptosis neurons and feeding to iMGLs. **c**. RT-qPCR quantification of cell cycle-associated genes after feeding (t-test, *p < 0.05, **p < 0.01, ***p < 0.001; n = 3 biological replicates).

To identify modulators of this signature, we used the Connectivity Map pipeline^46^, which contains gene expression data of nine human cell lines after thousands of perturbations, allowing association between a given transcriptomic signature and a specific alteration. This analysis revealed that genes dysregulated in microglia positively correlated with regulators of cell cycle and senescence, suggesting an exhaustion of microglial proliferation. We also found a negative correlation with a type I interferon-associated responses (*IFNB1*), often targeted in treatments for neurological diseases to reduce inflammation (Extended Data Fig. 10a).

By comparing our results with published snRNAseq studies^35,47^, we identified dysregulation of lipid metabolism (*APOE*, *APOC1* and *SPP1*) as a common feature in microglia, genes associated with DAMs shared between ALS and MS (*CTSD*, *GPNMB*, *CPM* and *LPL*) and ALS and AD (for example, *TREM2*), as inferred by bulk RNA sequencing studies^7^ (Fig. 5i). Genes specifically upregulated in ALS were related to vesicle trafficking, myeloid cell degranulation and the lysosome (for example, *SQSTM1/p62*, *LGALS3*, *GRN*, *ASAH1* and *LRRK2*). This evidence suggests the induction of a shared microglial reactive state, yet in ALS these changes are connected to endolysosomal pathways.

**Fig. 7:**
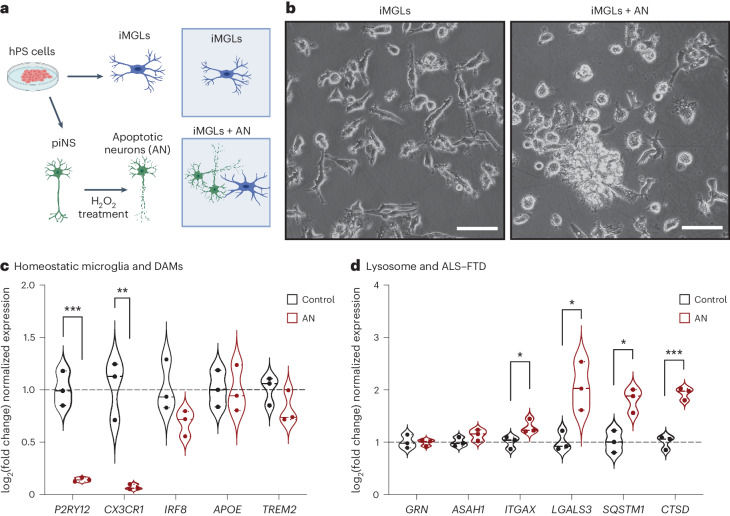
Apoptotic neurons upregulate lysosomal genes in microglia. **a**, Diagram of microglia and neuronal differentiation from PSCs and induction of apoptosis neurons and feeding to iMGLs (piNs, patterned induced neurons). **b**, Brightfield images of untreated day 40 iMGLs and day 40 iMGLs that were fed apoptotic neurons for 24 h. **c**. RT–qPCR quantification of homeostatic and DAM genes after feeding (AN, apoptotic neurons). **d**, RT–qPCR quantification of selected ALS–FTD-associated and lysosomal genes 24 h after feeding iMGLs with apoptotic neurons (AN, apoptotic neurons). (*t*-test, *P < 0.05, **P < 0.01, ***P < 0.001, *n* = 3 biological replicates). piNS, induced neurons from hPS cells.

Given the stress signature identified in neurons, we wondered whether these transcriptomic changes were driven by neuronal distress. We differentiated induced microglia-like cells (iMGLs)^48^ and neurons^38,49^ from hPS cells, triggered neuronal apoptosis and then introduced apoptotic neurons to iMGLs in vitro^48^ (Fig. 7a,b and Extended Data Fig. 10b). Quantitative assessment by quantitative reverse trascription polymerase chain reaction (RT–qPCR) confirmed the treatment lead to significant downregulation of homeostatic genes, upregulation of genes involved in endolysosomal trafficking (*CTSD*, *ITGAX*, *LGALS3* and *SQSTM1/p62*) and downregulation of actively cycling cells markers (Fig. 7c,d and Extended Data Fig. 10c), suggesting that changes identified in microglia from patients are, at least in part, a response to neuronal apoptosis.

## Discussion

A key question in the study of neurodegeneration is why certain cell types are more susceptible to different diseases. In this study, we identified the enrichment for ALS–FTD-associated genes in a class of ETNs, which provides a connection between this neuronal subtype and its propensity to accumulate TDP-43 aggregates^31^ leading to their gradual loss in ALS–FTD^10^. This enrichment is not recapitulated for risk factors connected to AD and MS, related to immune processes and more enriched in microglia^18–20^. One study suggested that ALS-associated variants connected to autophagy and protein clearing are most highly expressed in glutamatergic neurons^33^, and these findings add to the importance of axonal dynamics and ribonucleotide metabolism^34^; here, we provide a more detailed dissection of which subtype that might be.

Additionally, we identified a broadly shared transcriptomic signature of cellular stress pathways in classes of deep-layer excitatory neurons. These alterations in RNA translation and proteostasis have been implicated in models of ALS^1,2^; our study highlights their cell type specificity and links them to rare mutations in regulators of these pathways in familial forms of ALS^4^. These molecular mechanisms are confirmed to be connected to proteasomal function and proteostasis by human neuronal in vitro models, a system that is already being used to identify therapeutic candidates funneled into clinical trial pipelines^50^. The nuclear nature and the low coverage of this kind of sequencing methodology, but also the small sample size in our study, obviates further, confident dissection of the neuronal-specific changes in RNA biology identified in in vitro models and patient samples^37^. Moreover, the phenotypic and potentially genetic heterogeneity of sporadic ALS is probably reflected in our dataset and should be considered. This study is not powered to distinguish the relative contribution of technical effects versus intrinsic heterogeneity across individuals with sALS, but it opens the field to important considerations for future studies that should include greater numbers of patients. Nonetheless, our report highlights the importance of RNA metabolism and proteostasis and their specific misregulation in ETNs. Mouse models where these pathways are specifically altered in CSMNs might shed a light on their interplay in this specific neuronal type.

We suggest two mechanisms by which ETNs are rendered more susceptible to sALS: (1) the intrinsically higher expression of risk factors and (2) processes of degeneration in classes of ETNs that might exacerbate/contribute to their vulnerability in a combinatorial effect. Recent snRNAseq studies unraveled susceptibility of specific neuronal types in other diseases: mid-layer *RORB* neurons in AD^51,52^, upper-layer *CUX2* neurons in MS^22^, dopaminergic neurons in Parkinson’s disease^53^ and ETNs affected in ALS–FTD as described by our study and spinal cord motor neurons as recently suggested in ALS^54^. Impairment of proteostatic mechanisms seems to be a common theme in degenerating neurons regardless of the disease; however, only in ALS are these changes specifically connected to upregulation of transcripts connected to RNA metabolism, a trend that appears to go in the opposite direction in AD^52^. Integrative analyses of these studies might mark the beginning of a new understanding of the mechanisms behind selective neuronal vulnerability to different diseases.

Emerging studies have shown that glial cells are important modifiers in ALS–FTD^44^. We show that changes in processes involved in oligodendrocyte differentiation and myelination may contribute to degeneration and/or be a coordinated response to ALS and appear to contrast with those described in MS^43^. We revealed perturbations in key myelin regulators, such as *OPALIN*, *CNP* and *MAG*, across oligodendrocyte clusters but in these cells only, as opposed to AD where myelination-related changes were present across multiple cell types^35,55^. Given the similarities in the stress signature identified in neurons with changes in MS lesions but not in AD, it is puzzling how changes in myelination might be a consequence or cause of neuronal degeneration.

Intriguingly, recent work showed expression of neuronal RNA in oligodendrocytes in human motor cortex^24^. Upregulation of synaptic transcripts in this cell type in patients with sALS might represent phagocytic activity^56^ or the need for synaptic proteins during deposition of myelin sheath^57^. These speculations are interesting if coupled with the upregulation of synaptic machinery in upper layer neurons and the documented loss of postsynaptic molecules in ETNs in ALS^58^. Recent snRNAseq studies of FTD cortices identified changes in myelinating cells in response to neuronal loss and underlined the importance of cell-to-cell communication^40^, also GWAS studies have pointed at excitatory neurons, myelinating cells and inhibitory neurons’ sensitivity to genetic risks for ALS^34^. These observations suggest a coordinated response of the motor circuit in an attempt to compensate for loss of inputs to the cord. Further investigations could focus on shifting oligodendroglial states in mouse models and determine changes during disease progression to complement efforts aimed at controlling neuronal activity^59^.

Finally, we found distinct perturbations in ALS-associated microglia, particularly in endolysosomal pathways. We and others have implicated ALS–FTD-associated gene *C9orf72* in endosomal trafficking and secretion in myeloid cells^44,45^ and the upregulation of lysosomal constituents, for example, *CTSD*, was identified in this study and by others in patients^60^. Coupled with the upregulation of ALS–FTD-associated genes *SQSTM1/p62*, *OPTN*, *TREM2* and *GRN*, this suggests a mechanistic convergence on vesicle trafficking and inflammatory pathways that may initiate/exacerbate the homeostatic-to-DAM transition in ALS^7^. The interferon response-related changes we delineate, as identified by others in *C9orf72* ALS^61^, provide a parallel between sporadic and familial ALS. Overall, these changes had partial overlap with microglia in AD^35^ and in MS^47^, suggesting that drugs modulating myeloid cells in neurodegenerative diseases may provide a basis for new therapeutic approaches. Recent reports showed how DAM might be beneficial in disease contexts^62^, but others have inferred that microglial activation might result in poor disease outcomes. Studies manipulating microglial states might elucidate their ‘friend or foe’ role in sALS^48^.

In summary, we show that classes of ETNs require the expression of a collection of genetic risk factors for ALS–FTD with pivotal roles in proteostasis. This intrinsically higher expression of disease-associated genes might be at the bottom of a ‘first over the line’ mechanism leading to disruptions in groups of deep layer excitatory neurons. These alterations trigger a cascade of responses in glia: oligodendroglia shift from a myelinating to a neuronally engaged state and microglia activate a proinflammatory signature. Our study offers a view in which neurocentric disease vulnerability might spark responses in other cell types, but it also shows that enrichment of ALS–FTD-related genes in ETNs is coupled with processes engaging these genes in other cells too, that is, microglia. This view is a first insight into the disruptions of cortical biology in ALS and provides a connection between changes in cellular components and mechanisms associated with ALS. Future investigations should consider multicellular disruptions in ALS–FTD, where the survival of the neuron is unmistakably pivotal, but targeting other cells to reduce inflammation, promote myelination and bolster neuronal circuitry may re-establish a neuroprotective environment.

## Limitations of this study

One limitation is the small size of the cohort. ALS is a heterogeneous disease^7^ and smaller cohort sizes might not fully recapitulate its etiological diversity. Only recently have biobanks started to collect enough samples^6–8^, and we hope that increased sample availability and affordability of single-nucleus technology will allow a more comprehensive view of alterations in ALS. A larger cohort would also enable a more stringent analysis of differentially expressed transcripts that incorporates more sophisticated analytical tool.

We recognize that consensus on best practices in snRNAseq is still being reached, including for re-analysis of published studies^63,64^. We acknowledge that using single cells as a variable and not pseudobulked individuals might yield larger gene sets with possible confounding factors. We also recognize that our additional analyses revealed that some specific genes might be derived from outlier-driven effects and because of that we limited follow-up analyses only to genes shared by at least four out of five individuals. This is why we highlighted common features in wider biological pathways disrupted in cell types in patients with ALS followed up by validations at the protein level in a separate cohort of patients’ samples or in our in vitro studies. We hope that new reports will take into account the need for a more stringent investigation of reproducibility and we advocate for a more transparent conveying of results.

We also recognize that our study would benefit from additional validation at the RNA and/or protein level. This would elucidate some of the intriguing questions we raised. For example, are oligodendrocytes really expressing higher levels of neuronal genes or is this an artifact^65^? Nonetheless, we believe that this study provides novel insights in the involvement of different cell types in ALS and a different view in the motor cortex of patients with ALS. The increase in cohort sizes, more sophisticated analyses and new technologies, such as spatial transcriptomics, might further enrich the understanding of neurodegeneration in ALS.

## Methods

### Human donor tissue sources and ethics

Frozen postmortem human cortical samples from cases of patients with sporadic ALS and age-matched controls were obtained from the Target ALS Neuropathology Core that drew upon the repositories of five institutions. Specimens from the medial, lateral or unspecified motor cortex were grouped together. Deidentified postmortem brain tissue samples were obtained from the Massachusetts Alzheimer’s Disease Research Center at Massachusetts General Hospital (MGH) and the Target ALS Multicenter Human Postmortem Tissue Core, which integrates five academic tissue repositories for ALS research. The protocols of the Massachusetts Alzheimer’s Disease Research Center for brain donations and the collection of postmortem tissue and clinical information for research purposes were approved by the Institutional Review Board of Partners Healthcare (currently Mass General Brigham) at MGH. Informed consent for brain autopsy and the use of postmortem tissue for research was provided by the legal next-of-kin in compliance with local and institutional guidelines at all brain tissue repositories involved. Use of postmortem deidentified tissue samples followed ‘Not Human Subjects Research’ determinations by the Harvard Faculty of Arts and Sciences and Partners (MGH) and considered exempt from the instituational research board given lack of interaction with living individuals/participants. The study protocol was further approved by Harvard Stem Cell and Regenerative Biology Department, Harvard University. Informed consent and study protocol for human stem cell work were provided by Stanley Center for Psychiatric Research at Broad Institute of MIT and Harvard and the Harvard Stem Cell and Regenerative Biology Department at Harvard University.

### Isolation of nuclei

RNA quality of brain samples was assessed by running bulk nuclear RNA on an Agilent TapeStation for RNA integrity number scores. Extraction of nuclei from frozen samples was performed as previously described^66^. Briefly, tissue was dissected and minced with a razor blade on ice and then placed in 4 ml ice-cold extraction buffer (wash buffer (82 mM Na2SO4, 30 mM K2SO4, 5 mM MgCl2, 10 mM glucose and 10 mM HEPES, pH adjusted to 7.4 with NaOH) containing 1% Triton X-100 and 5% Kollidon VA64). Tissue was homogenized with repeated pipetting, followed by passing the homogenized suspension twice through a 26.5 gauge needle on a 3 ml syringe (prechilled), once through a 20 mm mesh filter and once through a 5 mm filter using vacuum. The nuclei were then diluted in 50 ml ice-cold wash buffer, split across four 50 ml tubes and centrifuged at 500*g* for 10 min at 4 °C. The supernatant was discarded, the nuclei pellet was resuspended in 1 ml cold wash buffer.

### 10× loading and library preparation

Nuclei were 4,6-diamidino-2-phenylindole (DAPI) stained with Hoechst, loaded onto a hemocytometer and counted using brightfield and fluorescence microscopy. The solution was diluted to ~176 nuclei μl^−1^ before proceeding with Drop-seq, as described^14^. Complementary DNA amplification was performed using around 6,000 beads per reaction with 16 PCR cycles. The integrity of both the complementary DNA and tagmented libraries were assessed for quality control on the Agilent Bioanalyzer. Libraries were sequenced on a Nova-seq S2, with a 60 bp genomic read. Reads were aligned to the human genome assembly (hg19). Digital gene expression files were generated with the Zamboni drop-seq analysis pipeline, designed by the McCarroll group^66,67^.

### Filtering of expression matrices and clustering of single nuclei

A single matrix for all samples was built by filtering any barcode with less than 400 genes and resulting in a matrix of 27,600 genes across 119,510 barcodes. This combined UMI matrix was used for downstream analysis using Seurat (v3.0.2)^15^. A Seurat object was created from this matrix by setting up a first filter of min.cells=20 per genes. After that, barcodes were further filtered by number of genes detected nFeature_RNA > 600 and nFeature_RNA < 6,000. Distribution of genes and UMIs were used as parameters for filtering barcodes. The matrix was then processed via the Seurat pipeline: log normalized by a factor of 10,000, followed by regressing UMI counts (nCount_RNA) and scaled for gene expression.

After quality filtering, 79,830 barcodes and 27,600 genes were used to compute shared nearest-neighbor graphs and *t*-distributed stochastic neighbor embedding (*t*SNE) projections using the first ten statistically significant principal components. As previously described^44,68^, *t*SNE projection was used to determine minimum number of clusters at Resolution=0.2 (FindClusters). Broad cellular identities were assigned to groups on the basis of DEGs as calculated by Wilcoxon rank sum test in FindAllMarkers(min.pct=0.25, logfc.threshold=0.25). One subcluster with a specifically high ratio of UMIs/genes was filtered out resulting in 79,169 barcodes grouped in seven major cell types. Markers for specific cell types were identified in previously published small conditional RNA sequencing studies^16^.

Analysis of cellular subtypes was conducted by subsetting each group. Isolated barcodes were renormalized and scaled and relevant principal components were used for clustering as a separate analysis. Newly scaled matrices were used for DGE analysis with MAST algorithm in Seurat, as previously reported^38,43,44,53,68^, with parameters FindAllMarkers(min.pct=0.10, logfc.threshold=0.25) and subclustering for identification of subgroups. DGEs for downsampled excitatory neurons groups were computed by adding parameters to the functions described above, FindAllMarkers(min.pct=0.10, logfc.threshold=0.25, min.cells = ‘minimum number in the group’, random.seed=TRUE), and re-iterative lists were generated with >95% of overlap (data not shown). PCA by individual was performed using matrices generated by AverageExpression(celltype, group.by = ”ID”, return.seurat=TRUE); these were normalized and scaled and PCA was run using the DGEs with RunPCA(celltype.averages, features=c(DGEs), npcs=7). To further confirm that changes in DGEs were not driven by one individual only, we ran DGE analysis by excluding cells from one ALS individual at a time (control versus ALS-1) in a re-iterative manner and showed that magnitude of changes in DGE expression were still similar and that identified DGEs were shared by all five or at least four out of five individuals; these gene lists were used for GOy and protein–protein interaction analyses. Gene scores for different cellular subclusters were computed in each renormalized, rescaled submatrix using the AddModuleScore function in Seurat v3.0.2.

Re-analysis of publicly available datasets was performed using matrices and metadata available. Only barcodes with available metadata concerning their cellular identity were selected to use identities assigned by peer review publication^22,30^. The available barcodes were then loaded into Seurat v4.0.1 (ref. ^69^). Gene scores for different cellular subclusters were computed in each renormalized, rescaled submatrix using the AddModuleScore function, as previously described. Re-analysis of spatial transcriptomic from Maynard et al. was performed using publicly available data and codes from publication itself^29^.

### GO, interactome and GSEAs

For GO terms analysis, we selected statistically significant upregulated or downregulated genes identified in each subcluster as described before (adjusted *P* values <0.05, log fold change (FC) of 2). These lists were fed in the gProfiler pipeline^70^ with the following settings: use only annotated genes, g:SCS threshold of 0.05, GO cellular components and GO biological processes (26 May 2020 to 9 December 2021), only statistically significant pathways are highlighted. Only statistically significant upregulated genes identified in each subcluster as described before (adjusted *P* values < 0.05, log FC of 2) were used for GO analysis. The interactome map was built using STRING^71^ protein–protein interaction networks, all statistically significant upregulated genes were used, 810 were identified as interacting partners using ‘experiments’ as interaction sources and a medium confidence threshold (0.400), only interacting partners are shown in Extended Data Fig. 6. Gene set enrichment analysis (GSEA) was performed using GSEA software designed by UC San Diego and the Broad Institute (v4.0.3)^72^. Briefly, gene expression matrices were generated in which for each subcluster each individual was a metacell, lists for disease-associated risk genes were compiled using available datasets (PubMed, ALS–FTD; Source Data Table 2) or recently published GWAS for AD^18,19^ and MS^20^.

### Generation of microglia-like cells

Microglial-like cells were differentiated as described^48^. Briefly, WA01-H1 hPS cells were cultured in E8 medium (Stemcell Technologies) on Matrigel (Corning), dissociated with accutase (Stemcell Technologies), centrifuged at 300*g* for 5 min and resuspended in E8 medium with 10 μM Y-27632 ROCK inhibitor, with 2 M cells transferred to a low-attachment T25 flask in 4 ml of medium and left in the suspension for 24 h. The first 10 days of differentiation are carried out in iHPC medium: Iscove’s modified Dulbecco’s medium (50%, Stemcell Technologies), F12 (50%, Stemcell Technologies), ITS-G-X 2% vol/vol (Thermo Fisher), l-ascorbic acid 2-phosphate (64 μg ml^−1^, Sigma), monothioglycerol (400 mM, Sigma), PVA (10 mg ml^−1^; Sigma), glutamax (1×, Stemcell Technologies), chemically defined lipid concentrate (1×, Stemcell Technologies) and nonessential amino acids (Stemcell Technologies). After 24 h (day 0), cells were collected, and differentiation is started in iHPC medium supplemented with fibroblast growth factor 2 (FGF2) (PeproTech, 50 ng ml^−1^), bone morphogenetic protein (PeproTech, 50 ng ml^−1^), activin A (PeproTech, 12.5 ng ml^−1^), Y-27632 ROCK inhibitor (1 μM) and LiCl (2 mM) and transferred in a hypoxic incubator (20% O_2_, 5% CO_2_ at 37 °C). On day 2, the medium was changed to iHPC medium plus FGF2 (PeproTech, 50 ng ml^−1^) and vascular enothelial growth factor (PeproTech, 50 ng ml^−1^) and returned to hypoxic conditions. On day 4, cells were resuspended in iHPC medium supplemented with FGF2 (PeproTech, 50 ng ml^−1^), vascular endothelial growth factor (PeproTech, 50 ng ml^−1^), TPO (PeproTech, 50 ng ml^−1^), SCF (PeproTech, 10 ng ml^−1^), interleukin (IL)-6 (PeproTech, 50 ng ml^−1^) and IL-3 (PeproTech, 10 ng ml^−1^), and placed into a normoxic incubator (20% O_2_, 5% CO_2_ at 37 °C). Expansion of hematopoietic progenitors was continued by supplementing the flasks with 1 ml of iHPC medium with small molecules every 2 days. On day 10, cells were collected and filtered through a 40 mm filter. The single-cell suspension was counted and plated at 500,000 cells per well in a six-well plate coated with Matrigel (Corning) in microglia differentiation medium: Dulbecco’s modified Eagle medium/F12 (Stemcell Technologies), ITS-G 2% vol/vol (Thermo Fisher Scientific), B27 (2% vol/vol, Stemcell Technologies), N_2_ (0.5% vol/vol, Stemcell Technologies), monothioglycerol (200 mM, Sigma), glutamax (1×, Stemcell Technologies), nonessential amino acids (1×, Stemcell Technologies), supplemented with macrophage colony-stimulating factor (25 ng ml^−1^, PeproTech), IL-34 (100 ng ml, PeproTech) and ransforming growth factor β-1 (50 ng ml^−1^, PeproTech). iMGLs were kept in this medium for 20 days with change three times a week. On day 30, cells were collected and plated on poly-d-lysine/laminin-coated dishes in microglia differentiation medium supplemented with CD200 (100 ng ml^−1^, Novoprotein) and CX3CL1 (100 ng ml^−1^, PeproTech), macrophage colony-stimulating factor (25 ng ml, PeproTech), IL-34 (100 ng/ml, PeproTech) and transforming growth factor β-1 (50 ng ml^−1^, PeproTech) until day 40.

### Feeding of apoptotic neurons to microglia-like cells

For feeding assays, neurons were generated from human iPSCs using an NGN2 overexpression system, as described previously^38,49,73^. Day-30 WA01-H1 hiPSC-derived neurons were treated with 2 μM H_2_O_2_ for 24 h to induce apoptosis. Apoptotic neurons were gently collected from the plate and the medium containing the apoptotic bodies was transferred into wells containing day 40 iMGLs. After 24 h, iMGLs subjected to apoptotic neurons and controls were collected for RNA extraction.

### RNA extraction and RT–qPCR analysis

RNA was extracted with the miRNeasy Mini kit (Qiagen, 217004). cDNA was produced with iScript kit (Bio-Rad) using 50 ng of RNA. RT–qPCR reactions were performed in triplicates using 20 ng of cDNA with SYBR Green (Bio-Rad) and were run on a CFX96 Touch PCR Machine for 39 cycles at 95 °C for 15 s, 60 °C for 30 s and 55 °C for 30 s.

### Generation of hiPSC-derived neurons for bulk RNA sequencing

Human embryonic stem cells were cultured in mTeSR (Stemcell Technologies) on Matrigel (Corning). Motor neurons were generated from HuES-3-Hb9:GFP based on the differentiation protocol previously described^74^. On completion of differentiation, cells were sorted via flow cytometry based on green fluorescent protein (GFP) signal intensity to yield GFP-positive neurons that were plated on PDL/laminin-coated plates (Sigma, Life Technologies). Neurons were maintained in neurobasal medium (Life Technologies) and supplemented with N_2_ (STEMCELL Technologies), B27 (Life Technologies), glutamax (Life Technologies), nonessential amino acids (Life Technologies) and neurotrophic factors (BDNF, GDNF and CNTF), and were grown for 28 days before the application of the proteasome inhibitors MG132 for 24 h.

RNA was extracted using RNeasy Plus kit (Qiagen), libraries were prepared using the Illumina TruSeq RNA kit v2 according to the manufacturer’s directions and sequenced at the Broad Institute core with samples randomly assigned between two flow chambers. The total population RNA sequencing FASTQ data was aligned against ENSEMBL human reference genome (build GRCh37/hg19) using STAR (v.2.4.0). Cufflinks (v.2.2.1) was used to derive normalized gene expression in fragments per kilo base per million. The read counts were obtained from the aligned BAM-files in R using Rsubread^73^. Differential gene expression was analyzed from the read counts in DESeq2 using a Wald’s test for the treatment dosage and controlling for the sequencing flow cell^73^.

### Western blot analysis

As previously described, tissue was minced, lysed in RIPA buffer with protease inhibitors (Roche) and sonicated^75^. After centrifugation, the supernatant was collected as soluble fraction and the insoluble pellet was resuspended in 8 M urea buffer (Bio-Rad, 1632103). After protein quantification by the bicinchoninic acid assay (Thermo Fisher), 10 µg of protein was preheated in Laemmli’s buffer (Bio-Rad) and loaded in 4–20% mini-PROTEAN TGX precast protein gels (Bio-Rad), and then gels were transferred to a polyvinylidene fluoride membrane. Membranes were blocked in Odyssey blocking buffer (Li-Cor) and incubated overnight at 4 °C with primary antibodies. After washing with TBS-T, membranes were incubated with IRDye secondary antibodies (1:10,000, Li-Cor) for 1 h and imaged with the Odyssey CLx imaging system (Li-Cor). Primary antibodies used 1:1,000 dilution: TDP-43 (PeproTech 10782-2-AP), GAPDH (Millipore cat. no. MAB374; CST 2118 (14C10)), MBP (Thermo Fisher PA-1-10008), CNP (Abcam ab6319(11-5b)), 20S (Enzo BML-PW8195-0025) and ubiquitin (CST 3936 T (P4D1)). IRDye provided by Licor was used at 1:10,000 dilution.

### Immunofluorescence assays

Cells were washed once with phosphate-buffered saline (PBS), fixed with 4% paraformaldehyde for 20 min, washed again in PBS and blocked for 1 h in 0.1% Triton in PBS with 10% donkey serum. Fixed cells were then washed and incubated overnight with primary antibodies at 4 °C. Primary antibody solution was washed and cells were subsequently incubated with secondary antibodies (1:2,000, AlexaFluor, Life Technologies) at room temperature for 1 h, washed with PBS and stained with DAPI. Primary antibodies used were Tuj1 (1:250, R&D, MAB1195) and TDP-43 (1:200, PeproTech 10782-2-AP). Images were analyzed using FIJI.

### Proteasome activity assay

Neurons were sorted in 96-well plates and, after 2 weeks of maturation, treated for 24 h. Cells were washed with 1× PBS, exposed to ProteasomeGlo (Promega, G8660) and incubated for 30 min at room temperature. Fluorescence was measured using a Cytation 3 reader (BioTek).

### Statistics and reproducibility

No statistical method was used to predetermine sample size. No statistical methods were used to predetermine sample sizes but our sample sizes are similar to those reported in previous publications^22,24,26,29,30,40,43,44,47,48,51,52,54,68^. No data were excluded from the analyses. The experiments were not randomized. The investigators were not blinded to allocation during experiments and outcome assessment. Data distribution was assumed to be normal but this was not formally tested. Data collection and analysis were not performed blind to the conditions of the experiments. Software used for analyses were Licor (Image Studio version 2.1), GraphPad Prism (version 7 and above), ImageJ (FIJI version 2.14) with Nikon NIS Elements version 4.0, Seurat version 3.0.2 and version 4.0.1, and GSEA version 4.0.3.

### Reporting summary

Further information on research design is available in the Nature Portfolio Reporting Summary linked to this article.

### Supplementary information

Reporting Summary

Supplementary Data 1 Graphical abstract and working model. Our study highlights cell type-specific changes in premotor/motor cortex of patients with sporadic ALS. Specifically, we identify upregulation of synaptic molecules in excitatory neurons of upper cortical layers, interestingly correlating to hyperexcitability phenotypes seen in patients. Moreover, excitatory neurons of the deeper layers of the cortex, which project to the spinal cord and are most affected by the disease, show higher levels of cellular stresses than other neuronal types. Correspondently, oligodendrocytes transition from a highly myelinating state to a more neuronally engaged state, probably to counteract stressed phenotypes seen in excitatory neurons. At the same time, microglia show a reactive state with specific upregulation of endolysosomal pathways.

### Source data

Source Data Unmodified western blots from all manuscripts.

Source Data Source data tables: (1) Patient information. (2) Disease-associated genes used for module scores. (3) Genes upregulated in each neuronal subtype in patients with ALS. (4) Genes used for oligodendrocytes module scores.

## Data Availability

Transcriptomic raw data have been deposited in the Gene Expression Omnibus database under accession number GSE226753. All other data supporting the findings of this study are available as source data files or from the corresponding author upon reasonable request. All other data analyzed from previously published sources will be available at publication references in the manuscript (for Schirmer et al. Sequence Read Archive (SRA), under accession number PRJNA544731 and NCBI Bioproject ID: 544731; for Velmeshev et al. Sequence Read Archive, accession number PRJNA434002; for Maynard et al. available via GitHub at https://github.com/LieberInstitute/HumanPilot and https://github.com/LieberInstitute/spatialLIBD).
