# Supplementary material for: Single-nucleus sequencing reveals enriched expression of genetic risk factors in extratelencephalic neurons sensitive to degeneration in ALS
Source: Nat Aging. 2024 Jun 21;4(7):984–97. doi: 10.1038/s43587-024-00640-0 (PMC11257952; doi:10.1038/s43587-024-00640-0)
Supplement: Supplementary file 2 — Graphical abstract and working model. Our study highlights cell type-specific changes in premotor/motor cortex of patients with sporadic ALS. Specifically, we identify upregulation of synaptic molecules in excitatory neurons of upper cortical layers, interestingly correlating to hyperexcitability phenotypes seen in patients. Moreover, excitatory neurons of the deeper layers of the cortex, which project to the spinal cord and are most affected by the disease, show higher levels of cellular stresses than other neuronal types. Correspondently, oligodendrocytes transition from a highly myelinating state to a more neuronally engaged state, probably to counteract stressed phenotypes seen in excitatory neurons. At the same time, microglia show a reactive state with specific upregulation of endolysosomal pathways. [file 43587_2024_640_MOESM2_ESM.pdf]

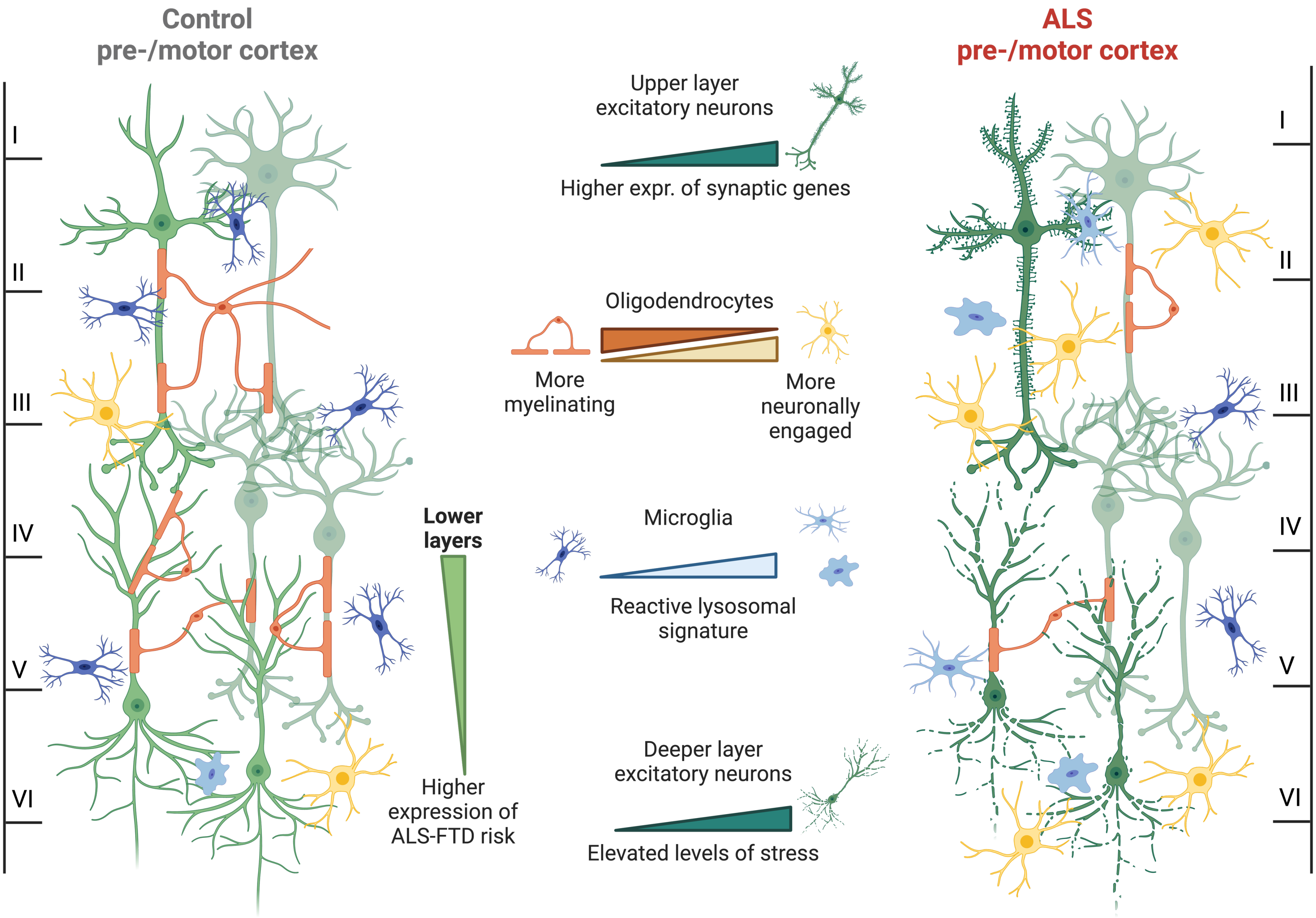

Control
pre-/motor cortex
ALS
pre-/motor cortex
I
II
III
IV
V
VI
Upper layer
excitatory neurons
Higher expr. of synaptic genes
Oligodendrocytes
More
myelinating
More
neuronally
engaged
Lower
layers
Higher
expression of
ALS-FTD risk
Microglia
Reactive lysosomal
signature
Deeper layer
excitatory neurons
Elevated levels of stress
