# Supplementary figures and images for: Single-nucleus sequencing reveals enriched expression of genetic risk factors in extratelencephalic neurons sensitive to degeneration in ALS
Source: Nat Aging. 2024 Jun 21;4(7):984–97. doi: 10.1038/s43587-024-00640-0 (PMC11257952; doi:10.1038/s43587-024-00640-0)

Fig. 2

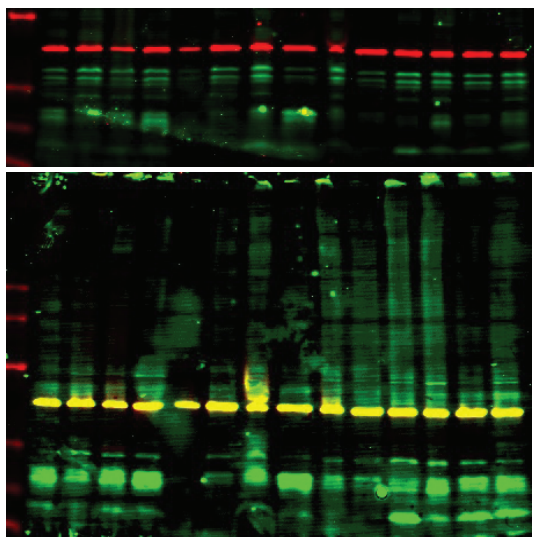

Fig. 4

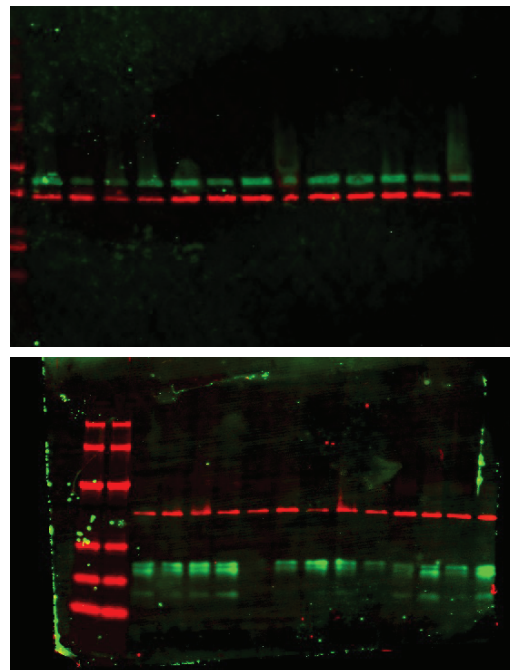

Extended Data Fig 6

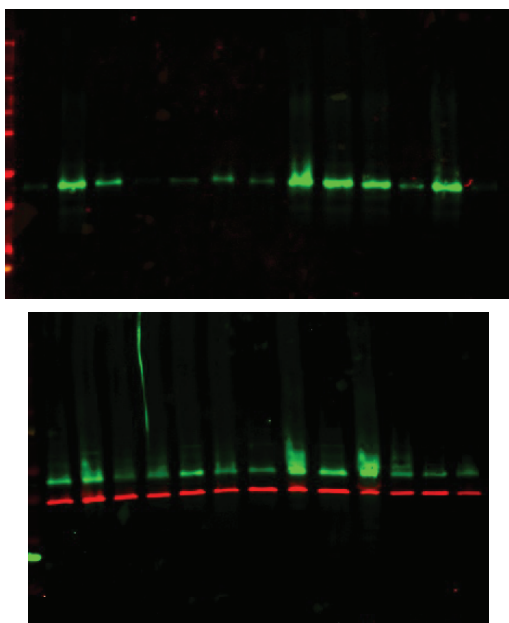

Supplement: Supplementary file 3 — Unmodified western blots from all manuscripts. [file 43587_2024_640_MOESM3_ESM.pdf]
